# Phenolics and Antioxidant Capacity of Basil (*Ocimum basilicum* L.) Genotypes Across Locations and Developmental Stages

**DOI:** 10.1007/s11130-026-01512-1

**Published:** 2026-05-13

**Authors:** Adem Zorlu, İsa Telci, Mahfuz Elmastaş, Oya Kaçar, Zehra Aytaç, Nusret Genç, Ömer Kayır

**Affiliations:** 1 Department of Phytotherapy, Hamidiye Institute of Health Sciences, University of Health Sciences, Üsküdar, Istanbul, 34668 Türkiye; 2 Department of Field Crops, Isparta University of Applied Sciences, Isparta, 32200 Türkiye; 3 Department of Field Crops, Faculty of Agriculture, Bursa Uludağ University, Bursa, 16059 Türkiye; 4 Department of Field Crops, Faculty of Agriculture, Eskişehir Osmangazi University, Eskişehir, 26040 Türkiye; 5 Department of Chemistry, Faculty of Arts and Sciences, Tokat Gaziosmanpaşa University, Tokat, 60150 Türkiye; 6 Scientific and Technical Application and Research Center, Hitit University, Çorum, 19030 Türkiye

**Keywords:** Antioxidant capacity, Chicoric acid, Dietary herb, HPLC–TOF, Rosmarinic acid, Sweet basil

## Abstract

Basil (*Ocimum basilicum* L.) is a globally consumed herb whose hydroxycinnamic acid esters and flavonoids contribute to dietary antioxidant intake. This study aimed to characterise the phenolic composition, antioxidant capacity, and developmental-stage variability of twelve Turkish basil genotypes grown across three ecologically contrasting sites. Twelve genotypes were evaluated in a two-year field experiment (Year 1 and Year 2) at three ecologically contrasting locations in Türkiye: Bursa, Eskişehir, and Tokat. Agronomic performance, nine-compound HPLC–TOF phenolic profiles (Year 1), total phenolic content (TPC), and antioxidant capacity (ABTS, DPPH, and FRAP) were measured. The effects of developmental stage on the phenolic composition of plants from Bursa (Year 1) were characterised. Location was the dominant source of variation in phenolic quality. Plants from Tokat ranked highest for individual phenolic concentrations (Year 1 HPLC–TOF data) and for all three antioxidant assays (two-year means). Rosmarinic acid varied 4.4-fold (59.0–261.8 mg 100 g⁻¹ DW; Year 1), with the highest values recorded for genotypes R-3k, R-19, R-15, and R-4 from Tokat. ABTS antioxidant capacity was 113% higher in plants from Tokat than in those from Bursa (two-year means: 483 vs. 227 µmol TE g⁻¹ DW). Analysis of rosmarinic acid in plants from Bursa showed an irregular, stage-dependent pattern, whereas TPC remained broadly stable across developmental stages (vegetative to full flowering: +3.9%). The genotype × stage interaction accounted for 44–50% of the total variance. PCA explained 89.3% of the variance across two components, with Tokat combinations separating clearly from those of the other sites. Under the tested field conditions, genotypes R-23 and R-4 emerged as strong candidates for phenolic-acid-targeted cultivation, while Y-15 recorded the highest two-year mean TPC at Tokat.

**Supplementary Information:**

The online version contains supplementary material available at 10.1007/s11130-026-01512-1.

## Introduction

Basil (*Ocimum basilicum* L.), a member of the Lamiaceae family, ranks among the world’s most widely cultivated aromatic herbs, valued both as a culinary ingredient and as a source of bioactive phytochemicals [1, 2]. Its leaves are rich in rosmarinic acid, chicoric acid, and hydroxycinnamic acid derivatives with documented antioxidant, anti-inflammatory, and antimicrobial properties [3–5]. Chicoric acid also acts as an inhibitor of HIV integrase [6, 7].

Türkiye spans a wide ecological gradient from the humid Marmara coast (Bursa, 95 m a.s.l.), through the high-altitude Central Anatolian steppe (Eskişehir, 800 m a.s.l.), to the transitional Black Sea zone (Tokat, 623 m a.s.l.), providing contrasting temperature, precipitation, radiation, and soil conditions that can influence phenolic accumulation in Lamiaceae [8–10]. Although genotype × environment studies on basil phenolics are available [4, 11, 12], many have relied on spectrophotometric total-phenolic assays without individual compound resolution or have covered only a limited number of sites. To our knowledge, no prior study has combined nine-compound HPLC–TOF phenolic profiling and three antioxidant assays across three ecologically distinct Turkish sites with a Bursa Year 1 developmental-stage assessment using a panel of local landraces and introduced cultivars. The present study aimed to: (i) quantify agronomic performance over two years; (ii) determine nine-compound phenolic profiles and TPC at all three locations; (iii) evaluate antioxidant capacity via ABTS, DPPH, and FRAP assays; and (iv) characterise developmental-stage effects on the phenolic composition of plants grown in Bursa.

## Materials and Methods

### Plant Material and Experimental Design

Twelve *Ocimum basilicum* L. genotypes were evaluated in a two-year field experiment across three ecologically distinct sites in Türkiye (Table 1) over two consecutive growing seasons (Year 1 = 2013; Year 2 = 2014). The study included ten Turkish landraces collected from multiple provinces across Türkiye between 2003 and 2010, as well as two commercial cultivars. Genotype characterisation is provided in Online Resource 1 (Table S1). Experiments were conducted using a randomised complete block design (RCBD) with three replications. Each plot measured 3.6 m², with 40 × 30 cm spacing. Seedlings were raised under greenhouse conditions and transplanted in late May. Irrigation and fertilisation were managed uniformly across all plots in accordance with standard local practice.

Plant height was recorded per plot as the mean of the first (flowering onset) and second (full flowering) harvests in each season. Measurements were taken from ten representative plants per plot at each harvest. The location-specific values reported in Table 2 represent two-year means, calculated by averaging the seasonal means from Year 1 and Year 2.

### Agronomic Measurements

Plant height (cm) was measured as described above (ten representative plants per plot per harvest). Fresh herb yield (FHY, kg ha⁻¹) was determined as total shoot mass per plot at flowering onset (5–10% open flowers).

### Developmental Stage Sampling

Developmental-stage sampling was restricted to Bursa in Year 1 to enable destructive harvesting within the available replicate structure. Leaf samples were collected at three developmental stages: (i) vegetative (V; no flower buds visible), (ii) flowering onset (FO; 5–10% open flowers), and (iii) full flowering (FF; >80% bloom). The three stages were sampled approximately 30–35, 50–55, and 65–75 days after transplanting, respectively, spanning early July to late August. Within each replicate block, separate plot units were assigned to each developmental stage, allowing destructive harvesting with independent observations. A composite sample (approximately 5 g fresh weight) was collected from all plants within each plot unit, immediately frozen at − 80 °C, lyophilised, and ground to a fine powder.

### Phenolic Profile Determination

Phenolic compounds were extracted using 70% (v/v) aqueous methanol (0.1 g in 10 mL) in an ultrasonic bath for 14 min at 14 °C. Extracts were screened against a 22-compound reference standard panel; nine compounds were detected and quantified in basil samples using HPLC–TOF (Agilent 1200/Bruker MicrOTOF): 4-hydroxybenzoic acid, ferulic acid, gallic acid, gentisic acid, caffeic acid, chicoric acid, rosmarinic acid, quercetin, and rutin (Sigma-Aldrich, Steinheim, Germany; purity ≥ 98%). The conditions for chromatography were as follows: ZORBAX SB-C18 column (150 × 4.6 mm, 3.5 μm); mobile phase A, 0.1% formic acid in water; mobile phase B, acetonitrile; gradient elution, 0–1 min 10% B, 1–20 min 10–50% B, 20–25 min 50–90% B, and 25–28 min 90% B; flow rate, 0.8 mL min⁻¹; injection volume, 10 µL; column temperature, 30 °C. Compounds were identified based on retention time and accurate mass in [M − H]⁻ mode. External calibration used seven concentration levels (25–2,500 ppb; r² = 0.985–0.997). Method validation parameters (LOD: 1.83–3.77 µg L⁻¹; LOQ: 5.55–11.42 µg L⁻¹) are provided in Online Resource 1 (Table S8). The sample injection order was randomised within each batch, and a calibration check standard was injected every 12 samples to monitor instrument drift. Peak-area repeatability across check standards was within ± 5% throughout the analytical campaign. Solvent blanks injected between sample batches confirmed the absence of carryover for all nine quantified compounds. Results are expressed as milligrams per 100 g of dry weight (mg 100 g⁻¹ DW). Samples analysed across locations (Year 1, all three sites) were collected at the FO stage, consistent with the FHY harvest stage. HPLC–TOF data from Year 2 failed pre-submission quality control and were therefore excluded; accordingly, individual phenolic profiles are reported only for Year 1.

### Total Phenolic Content and Antioxidant Capacity

TPC was determined using the Folin–Ciocalteu method [13] at all three locations in both years, with results expressed as milligrams of gallic acid equivalent *per* gram of dry weight (mg GAE g⁻¹ DW). Antioxidant capacity was measured using the DPPH [14], ABTS [15], and FRAP [16] assays, calibrated against Trolox and expressed as micromoles of Trolox equivalent per gram of dry weight (µmol TE g⁻¹ DW). Reagents used included Folin–Ciocalteu reagent and iron(III) chloride hexahydrate (Merck, Darmstadt, Germany), ABTS, potassium persulphate, DPPH, and TPTZ (Sigma-Aldrich).

### Statistical Analysis

A three-way ANOVA (genotype × location × year) was performed using SAS v9.4 (SAS Institute, Cary, NC, USA) with PROC GLM and Type III sums of squares. Mean separation was performed using Tukey’s HSD test, and compact-letter displays in Tables 3 and 4 were assigned at α = 0.05. Developmental-stage effects were evaluated for samples collected in Bursa using two-way ANOVA (genotype × stage), with stage units treated as independent observations within each block. Assumptions of residual normality and homogeneity of error variances were evaluated through visual inspection of residual-versus-fitted and Q-Q plots generated in SAS PROC GLM, supplemented by examination of cell-wise standard deviations reported in Online Resource 1 (Tables S2–S6). Bartlett’s test applied to the cell-wise variances within each location × year stratum did not detect significant heterogeneity (*p* > 0.05) for any of the four response variables (TPC, ABTS, DPPH, and FRAP), supporting the assumption of approximately homogeneous error variances across genotypes. Accordingly, no data transformations were applied prior to analysis. PCA was performed on z-scored genotype–location mean values of six variables (*n* = 36): rosmarinic acid (Year 1), chicoric acid (Year 1), TPC (two-year mean), ABTS (two-year mean), DPPH (two-year mean), and FRAP (two-year mean). Three-way ANOVA summaries are provided in Online Resource 1 for phenolic and antioxidant variables (Table S12) and for agronomic variables (plant height and FHY; Table S14). The two-way ANOVA summary for the Bursa developmental-stage experiment is presented in Table S13.

## Results and Discussion

### Site Climate Characteristics

Growing-season climate conditions are summarised in Table 1. The three sites differed substantially in thermal regime, moisture supply, radiation receipt, and soil characteristics. Bursa was the warmest (Year 1: 21.7 °C; Year 2: 22.0 °C) and most humid site (RH 58–64%), consistent with its low-altitude Marmara location. Eskişehir, located at the highest altitude (800 m a.s.l.), received the highest solar radiation (24.18 MJ m⁻² d⁻¹ in Year 1) but the lowest Year 1 precipitation (62 mm), with intermediate temperatures. Tokat exhibited the lowest temperature (Year 1: 19.3 °C), the lowest solar radiation (Year 1: 22.26 MJ m⁻² d⁻¹; two-year mean: 22.12 MJ m⁻² d⁻¹), and the highest soil organic matter content (2.3% vs. 1.4–2.1%), resulting in a distinct abiotic stress profile relative to the other two sites. Precipitation in Year 2 was higher at all sites, most markedly at Bursa (+ 115%) and Eskişehir (+ 98%), with a modest increase at Tokat (+ 19%). By contrast, mean temperatures and solar radiation remained broadly consistent across years. Inter-annual variation in precipitation was therefore the principal source of year-to-year environmental variability.

Climate data were obtained from the NASA POWER MERRA-2 reanalysis archive to ensure methodological consistency across locations and throughout the growing season. Local meteorological station readings (Turkish State Meteorological Service, MGM) for the same period may differ marginally from the satellite-derived values reported here, owing to spatial averaging in the reanalysis product.

**Table 1:** Characteristics of the three experimental sites

Parameter	Bursa	Eskişehir	Tokat
Latitude (°N)	40.18	39.78	40.31
Longitude (°E)	29.06	30.52	36.55
Altitude (m a.s.l.)	95	800	623
Soil texture	Clay loam	Sandy loam	Loam
Soil pH / OM (%)	7.2 / 2.1	7.6 / 1.4	7.0 / 2.3
GS temp. — Year 1 (°C)	21.7	20.1	19.3
GS temp. — Year 2 (°C)	22.0	20.7	20.8
GS precip. — Year 1 (mm)	78	62	68
GS precip. — Year 2 (mm)	168	123	81
GS RH — Year 1 (%)	58.1	56.6	60.2
GS RH — Year 2 (%)	63.5	62.2	61.1
GS solar — Year 1 (MJ m⁻² d⁻¹)	23.78	24.18	22.26
GS solar — Year 2 (MJ m⁻² d⁻¹)	22.22	22.99	21.97

GS: June–September growing season. Temperature, precipitation, relative humidity: NASA POWER MERRA-2; solar radiation: CERES SYN1deg. *OM* organic matter

### Agronomic Performance

Plant height and FHY are presented in Table 2, and Fig. S1 provides a graphical summary. Genotype, location, and year each had significant main effects (*p* < 0.01). Plant height ranged from 26.0 to 64.4 cm in Year 1; R-10 A was consistently the tallest (two-year cross-location mean: 61.6 cm), while R-15 was the shortest (Year 1 mean: 35.4 cm). Location means ranked Tokat ≥ Bursa > Eskişehir. FHY ranged from 3,870 to 25,400 kg ha⁻¹, with location means of 13,346 kg ha⁻¹ (Tokat), 12,182 kg ha⁻¹ (Bursa), and 8,418 kg ha⁻¹ (Eskişehir). The FHY response to inter-annual climatic variation was genotype-specific. In Year 2, six of twelve genotypes showed higher yields (R-16: +31.2%, R-20: +25.4%, R-3k: +22.1%, R-10 A: +18.5%, Y-15: +14.7%, and R-19: +9.6%), whereas six showed lower yields (R-23: −14.8%, R-15: −12.6%, R-1: −8.4%, Y-7: −6.3%, R-4: −4.2%, and R-17: −2.1%). The cross-location grand mean increased modestly from 10,970 kg ha⁻¹ in Year 1 to 11,660 kg ha⁻¹ in Year 2 (+ 6.3%). This pattern paralleled genotype climate-of-origin: landraces collected from high-precipitation Black Sea provinces (R-16 Rize, R-20 Ordu, and R-3k Samsun) responded positively to the wetter conditions in Year 2, whereas landraces from semi-arid inland transitional zones (R-15 Tokat and R-23 Zonguldak) declined under the same increase in precipitation. R-10 A and R-17 were the highest-yielding genotypes across both years, consistent with their above-average plant height (Table 2). Canopy traits beyond plant height (e.g., leaf area index and branching pattern) were not recorded in this field trial.

**Table 2:** Plant height (cm) and fresh herb yield (FHY, kg ha⁻¹) of twelve *Ocimum basilicum* L. genotypes at three ecological sites in Türkiye (two-year means)

Genotype	Plant height (cm)					Fresh herb yield (kg ha⁻¹)				
	Bursa	Esk.	Tokat	Y1 mean	Y2 mean	Bursa	Esk.	Tokat	Y1 mean	Y2 mean
R-1	36.5	28.5	45.5	36.8	36.1	5,340	3,870	9,215	6,411	5,872
R-3k	48.7	48.6	57.1	51.5	50.8	11,450	9,700	13,584	10,426	12,730
R-4	45.1	38.0	53.2	46.0	44.8	10,950	9,320	13,098	11,361	10,884
R-10 A	61.1	60.5	63.2	62.0	61.2	25,400	11,850	19,201	17,224	20,410
R-15	37.2	33.7	35.4	35.4	35.0	8,090	7,580	9,626	8,999	7,865
R-16	45.3	43.8	51.0	46.4	47.0	7,300	7,200	15,946	8,779	11,518
R-17	52.0	48.5	57.8	52.6	53.3	15,950	10,600	17,814	14,945	14,631
R-19	46.3	43.6	52.3	47.4	47.6	8,500	7,950	12,061	9,068	9,939
R-20	42.8	38.6	47.5	43.0	43.1	11,400	8,700	12,036	9,505	11,919
R-23	49.5	46.2	55.0	49.8	51.1	10,300	7,650	11,603	10,638	9,064
Y-7	56.8	55.3	63.4	58.8	58.3	17,200	8,400	12,686	13,177	12,347
Y-15	44.5	40.1	43.6	42.7	43.1	14,300	8,200	13,283	11,111	12,744

Two-year means by location (Bursa, Eskişehir, Tokat; *n* = 6 plots per genotype–location). Y1 mean and Y2 mean = cross-location means for Year 1 and Year 2, respectively. Esk. = Eskişehir. Location-specific two-year means are graphically presented in Fig. S1.

### Individual Phenolic Profile Across Locations

Nine phenolic compounds were quantified using HPLC–TOF at all three locations in Year 1. Rosmarinic acid and chicoric acid data are presented in Table 3; the complete nine-compound dataset is provided in Online Resource 1 (Table S2).

Rosmarinic acid was the principal phenolic constituent in all samples [4, 5, 12], with a 4.4-fold range across genotype–location combinations (59.0–261.8 mg 100 g⁻¹ DW). The location ranking was Tokat > Eskişehir > Bursa (*p* < 0.01; Year 1 means: 189.7, 179.6, and 103.7 mg 100 g⁻¹ DW, respectively), a pattern in line with reports that abiotic conditions can influence PAL-associated phenolic biosynthesis in Lamiaceae [5, 9, 10]. The highest individual values were recorded for R-3k (261.8 mg 100 g⁻¹ DW at Tokat), R-19 (251.4 mg 100 g⁻¹ DW), R-15 (242.5 mg 100 g⁻¹ DW), and R-4 (240.2 mg 100 g⁻¹ DW). Significant G × L interactions (*p* < 0.01) indicate that site-specific genotype selection is needed [12, 17, 18].

Chicoric acid followed the same location trend (Tokat > Eskişehir > Bursa). The highest value was recorded for R-23 at Tokat (68.6 mg 100 g⁻¹ DW), followed by R-19 (59.6 mg 100 g⁻¹ DW), R-15 (50.3 mg 100 g⁻¹ DW), and R-3k (48.0 mg 100 g⁻¹ DW) [19]. Among the minor phenolics, rutin showed the widest variation (0.01–39.6 mg 100 g⁻¹ DW), indicating potential for the parallel breeding of both flavonoid and hydroxycinnamic acid fractions [10, 12]. The highest accumulation (R-23 at Tokat, 39.6 mg 100 g⁻¹ DW) is nutritionally relevant, given rutin’s reported capillary-protective and anti-inflammatory activity. Caffeic acid ranged from 0.26 to 0.86 mg 100 g⁻¹ DW; gallic, gentisic, ferulic, and 4-hydroxybenzoic acids were present at trace levels (< 0.5 mg 100 g⁻¹ DW) throughout [18, 20] and did not show consistent genotype- or location-driven enrichment.

**Fig. 1:**
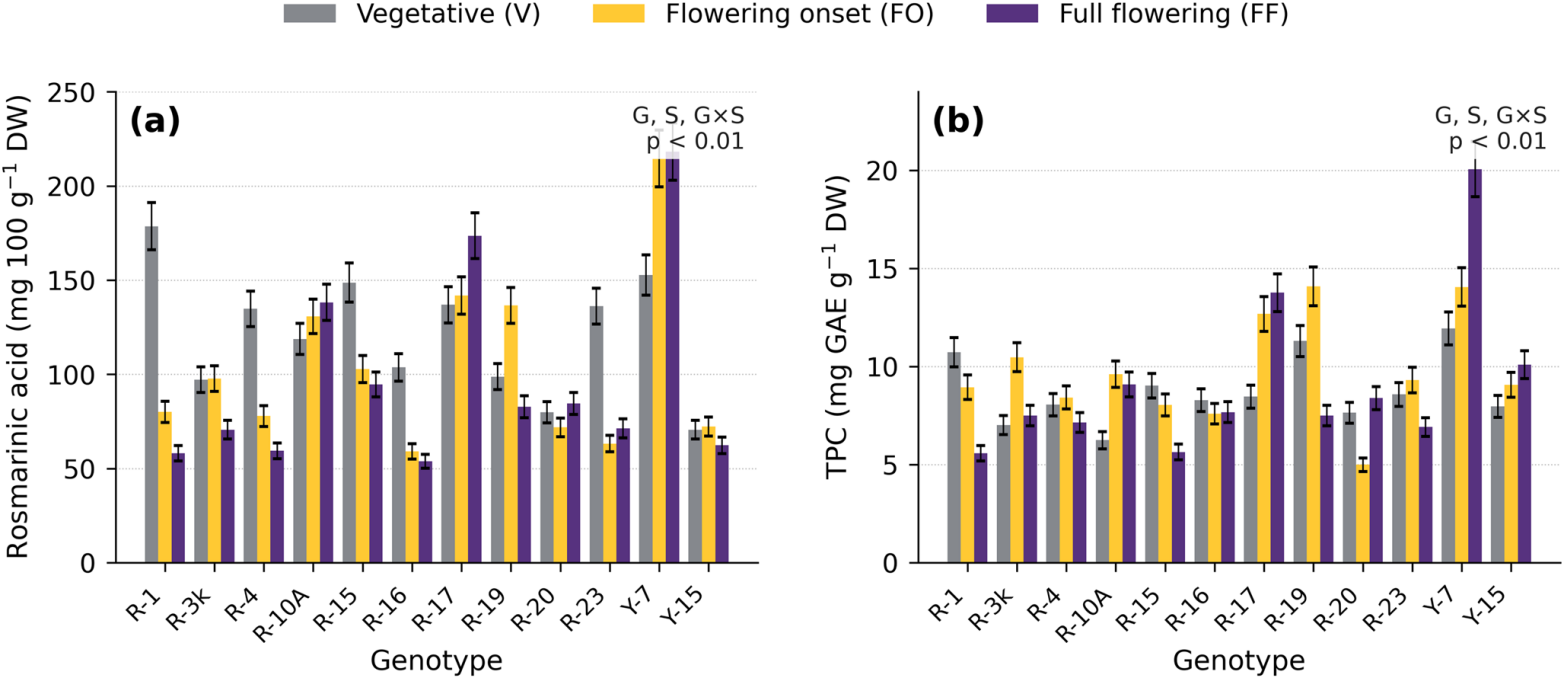
Rosmarinic acid (RA, mg 100 g⁻¹ DW; panel **a**) and total phenolic content (TPC, mg GAE g⁻¹ DW; panel **b**) of twelve *Ocimum basilicum* L. genotypes at three developmental stages, collected at the Bursa site in Year 1. The cross-genotype mean RA declined from vegetative (V) to full flowering (FF) (− 19.9%), while individual genotype responses were heterogeneous (in five genotypes, the FO or FF maximum exceeded the vegetative-stage value by more than 5%; see text). Cross-genotype mean TPC remained generally stable (+ 3.9%). Error bars show ± SD (*n* = 3 RCBD replicate plots). V, vegetative; FO, flowering onset; FF, full flowering

The absolute concentrations of rosmarinic acid (maximum 2.62 mg g⁻¹ DW) and chicoric acid (maximum 0.69 mg g⁻¹ DW; 68.6 mg 100 g⁻¹ DW) in this study fall within the lower-to-middle range reported for basil. The maximum rosmarinic acid value is within the 0.10–6.09 mg g⁻¹ DW range reported by Kwee and Niemeyer [21] for 15 commercial basil cultivars, falls within the lower part of the 1.31–21.31 mg g⁻¹ DW range reported by McCance et al. [22] for purple basil cultivars across maturity stages, and is markedly below the 5.41–47.89 mg g⁻¹ DW range reported by Nguyen and Niemeyer [18] for sweet basil grown under varied hydroponic nitrogen regimes. The maximum chicoric acid value (68.6 mg 100 g⁻¹ DW) is within the 6.48–242.50 mg 100 g⁻¹ DW range documented by Lee and Scagel [19] for fresh and dried commercial basil products. The comparatively moderate values in the present panel, particularly relative to hydroponically cultivated and purple-basil comparators, likely reflect three factors: (i) the predominantly Turkish landrace background of the material (10 of 12 entries are open-pollinated landraces from Black Sea and inland transitional provinces of Türkiye; Table S1), selected mainly for agronomic adaptation rather than maximum phenolic accumulation; (ii) the use of a single-step methanolic extraction without sequential solvent partitioning or matrix-matched recovery correction, which may give conservative quantitative estimates compared with optimised multi-step protocols; and (iii) the use of flowering-onset material for the across-location comparison, matching the FHY harvest stage, because the Bursa developmental-stage experiment showed flowering-onset rosmarinic acid to be 14.3% lower on average than vegetative-stage values (Fig. 1a; cross-genotype mean V = 121.4 → FO = 104.0 mg 100 g⁻¹ DW). These considerations do not alter the relative location- and genotype-driven patterns that support the main conclusions, but they should be considered when comparing absolute concentrations across studies.

**Table 3:** Rosmarinic acid and chicoric acid concentrations (mg 100 g⁻¹ DW, Year 1) of twelve *Ocimum basilicum* L. genotypes at three Turkish ecological sites. The complete nine-compound dataset is provided in Online Resource 1 (Table S2)

Compound	Location	*R*-1	*R*-3k	*R*-4	*R*-10 A	*R*-15	*R*-16	*R*-17	*R*-19	*R*-20	*R*-23	Y-7	Y-15
Rosmarinic acid	Bursa	78.3 d	97.2 ^*c*^	77.4 ^*d*^	130.8 ^*b*^	102.8 ^*c*^	59.0 ^*e*^	139.6 ^*b*^	137.6 ^*b*^	72.6 ^*de*^	62.0 ^*e*^	213.8 ^*a*^	73.5 ^*de*^
	Eskişehir	183.7 ^*cde*^	143.5 ^*f*^	181.8 ^*de*^	226.1 ^*a*^	198.4 ^*bcd*^	168.7 ^*e*^	206.1 ^*ab*^	170.0 ^*e*^	69.2 ^*g*^	205.0 ^*b*^	203.8 ^*bc*^	198.7 ^*bcd*^
	Tokat	125.5 ^*e*^	261.8 ^*a*^	240.2 ^*a*^	152.1 ^*d*^	242.5 ^*a*^	90.5 ^*f*^	198.8 ^*bc*^	251.4 ^*a*^	202.4 b	195.6 ^*bc*^	135.9 de	179.5 ^*c*^
Chicoric acid	Bursa	16.9 ^*d*^	19.8 ^*bcd*^	19.7 ^*bcd*^	20.1 ^*bc*^	17.7 ^*cd*^	7.9 ^*e*^	20.7 ^*ab*^	23.5 a	23.4 ^*a*^	21.9 ^*ab*^	17.5 ^*cd*^	9.0 ^*e*^
	Eskişehir	18.9 ^*d*^	17.1 ^*d*^	19.2 ^*d*^	23.3 ^*c*^	29.8 ^*b*^	13.4 ^*e*^	23.6 ^*c*^	23.8 ^*c*^	7.4 f	37.3 ^*a*^	16.0 ^*de*^	13.0 ^*e*^
	Tokat	25.7 fg	48.0 ^*c*^	41.7 ^*d*^	26.7 ^*fg*^	50.3 ^*c*^	13.0 ^*h*^	33.5 ^*e*^	59.6 ^*b*^	28.1 ^*ef*^	68.6 ^*a*^	21.7 ^*g*^	22.1 ^*g*^

Year 1 means (mg 100 g⁻¹ DW, *n* = 3 plot replicates). Within each row (location × compound), means followed by the same superscript letter do not differ significantly (Tukey’s HSD, α = 0.05). Compact-letter displays were generated from Tukey’s HSD post hoc comparisons of the corresponding SAS PROC GLM model. The complete nine-compound dataset with means ± SD is provided in Online Resource 1 (Table S2); a graphical visualisation is presented in Fig. S2

### Effect of Developmental Stage on Phenolic Composition

Developmental stage had significant (*p* < 0.01) effects on rosmarinic acid and TPC at Bursa (Fig. 1; Online Resource 1, Tables S9–S11 for data, Table S13 for ANOVA summary). Variance partitioning indicated that genotype and genotype × stage interactions accounted for a greater proportion of the total variance (43–50% and 44–50%, respectively) than stage main effects (5.7–6.4%; Table S13). Genotypic rankings for phenolic quality were largely preserved across developmental stages, consistent with cultivar-dependent maturity responses and genotype × harvest interactions reported in basil [21–24].

Rosmarinic acid exhibited an irregular, stage-dependent pattern in samples collected at Bursa (Year 1). In five genotypes (R-10 A, R-17, R-19, R-20, and Y-7), the maximum RA concentration at flowering onset (FO) or full flowering (FF) exceeded the vegetative-stage value by more than 5%, whereas the remaining seven genotypes peaked at or near the vegetative stage [22, 25]. Across genotypes, the mean RA concentration declined by 19.9%, from 121.4 mg 100 g⁻¹ DW at the vegetative stage to 97.3 mg 100 g⁻¹ DW at FF. RA concentration was highest in R-1 at the vegetative stage (178.6 mg 100 g⁻¹ DW) and in Y-7 at FF (218.3 mg 100 g⁻¹ DW). TPC remained generally stable across stages (+ 3.9%), although pronounced increases were observed at FF in Y-7 (20.1 mg GAE g⁻¹ DW) and R-17 (13.8 mg GAE g⁻¹ DW). The contrasting trajectories of RA and TPC suggest a stage-dependent shift in the relative contribution of rosmarinic acid to the Folin-reactive phenolic pool [5, 9, 22]. The RA/TPC ratio (milligram of rosmarinic acid per milligram of GAE) declined from 0.139 at the vegetative stage to 0.107 at FF (a reduction of approximately 23%). Thus, rosmarinic acid contributed a smaller share of the Folin-reactive phenolic pool at full flowering, although broader targeted or untargeted profiling would be needed to identify the compound classes responsible for this shift. In practical terms, harvesting at the vegetative stage may favour RA accumulation, whereas harvesting at FF may maximise TPC in high-accumulating genotypes. However, these patterns reflect site-specific dynamics at Bursa (Year 1), and multi-location, multi-year validation is required before stage-specific recommendations can be applied in practice [20, 22, 23].

### Total Phenolic Content and Antioxidant Capacity

TPC and antioxidant capacity are presented in Table 4. TPC ranged from 6.4 to 18.6 mg GAE g⁻¹ DW (two-year means). The location ranking was consistent: Tokat > Eskişehir > Bursa (*p* < 0.01; means: 15.4, 13.0, and 8.1 mg GAE g⁻¹ DW, respectively). The three-way ANOVA (Online Resource 1, Table S12) showed that location accounted for 46.3% of total TPC variance (SS%), while the year effect was non-significant (*p* = 0.165), indicating inter-annual stability of the location ranking. The highest two-year mean TPC was recorded in Y-15 at Tokat (18.6 mg GAE g⁻¹ DW), followed by R-3k (18.0 mg GAE g⁻¹ DW), R-10 A (17.9 mg GAE g⁻¹ DW), Y-7 (17.8 mg GAE g⁻¹ DW), and R-23 (17.0 mg GAE g⁻¹ DW).

The higher phenolic content in samples from Tokat coincided with lower mean solar radiation (22.12 MJ m⁻² d⁻¹ at Tokat compared with 23.59 MJ m⁻² d⁻¹ at Eskişehir), lower two-year mean precipitation (75 mm at Tokat vs. 93 and 123 mm at Eskişehir and Bursa, respectively), and the highest soil organic matter content (2.3 vs. 1.4–2.1%). These environmental differences are consistent with reports of enhanced phenolic accumulation under moderate abiotic stress in Lamiaceae [5, 9, 10, 26]. However, the present study did not measure PAL activity, gene expression, or carbon-flux parameters; therefore, the underlying biosynthetic mechanisms remain hypothetical. Causal relationships cannot be inferred from observational field data collected at only three locations.

**Fig. 2:**
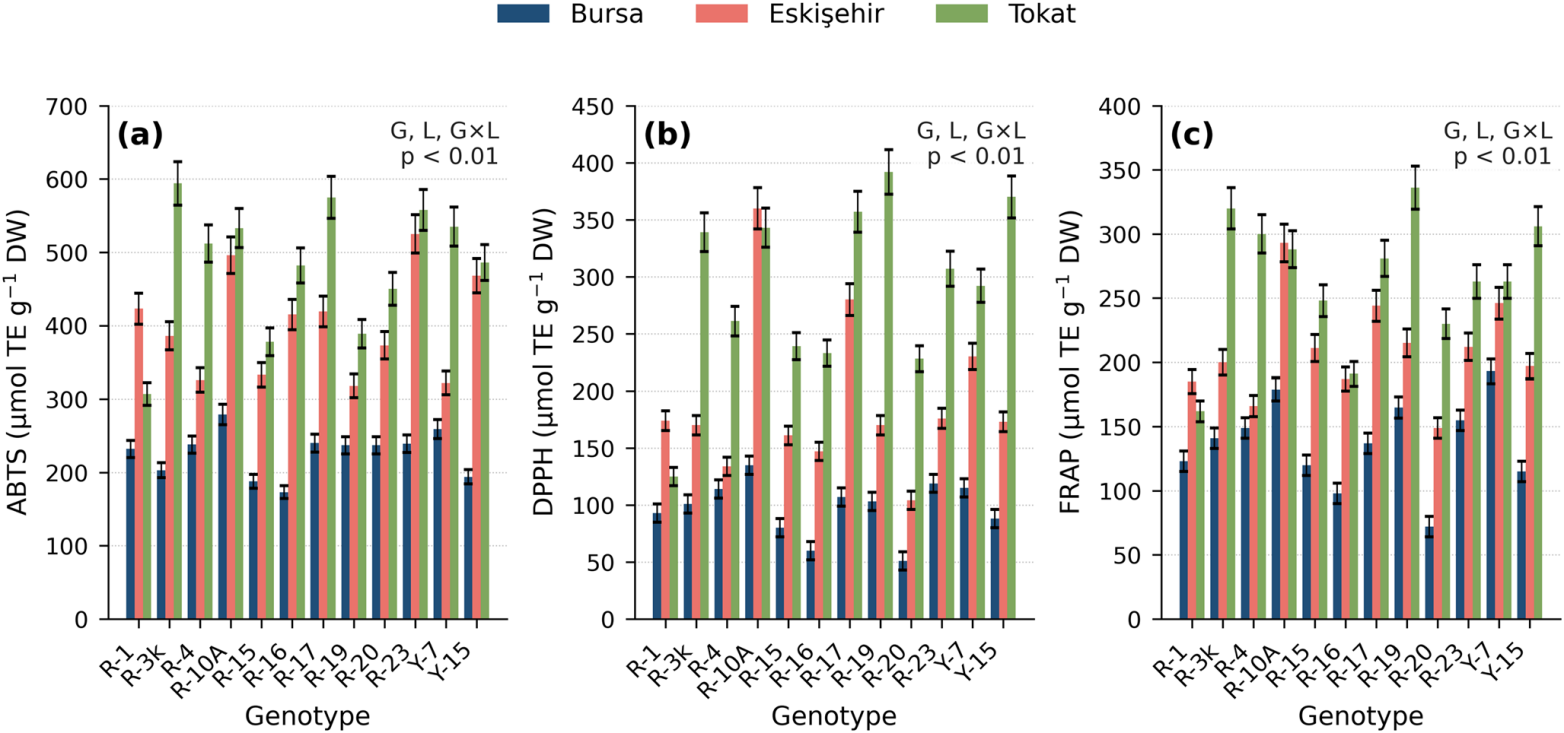
ABTS (**a**), DPPH (**b**), and FRAP (**c**) antioxidant capacity (µmol TE g⁻¹ DW) of twelve basil genotypes at three Turkish ecological sites (two-year means). Error bars = ± SD (*n* = 6 RCBD replicate plots *per* genotype–location)

All three antioxidant assays showed a consistent ranking of locations: Tokat > Eskişehir > Bursa (*p* < 0.01). ABTS, DPPH, and FRAP genotype-by-location patterns are shown in Fig. 2 (location means: ABTS 227 / 400 / 483; DPPH 97 / 190 / 291; FRAP 137 / 209 / 266 µmol TE g⁻¹ DW for Bursa / Eskişehir / Tokat, respectively). The year effect was significant for ABTS (*p* < 0.001; Year 2 + 34.0%), DPPH (*p* < 0.001; Year 2 + 76.3%), and FRAP (*p* < 0.001; Year 2 − 4.7% overall, with site-dependent direction: Bursa − 18.9%, Eskişehir + 4.2%, Tokat − 3.5%; Table S6). The increases in ABTS and DPPH coincided with higher Year 2 precipitation at all sites, although causality cannot be inferred from the present field design [9, 24, 27]. The divergent FRAP response may partly reflect assay-mechanism selectivity: FRAP measures single-electron-transfer (Fe³⁺ reduction) capacity, whereas ABTS and DPPH can also capture hydrogen-atom-transfer activity [28]. Year-to-year shifts in phenolic subclass composition could therefore influence the three assays differently even when TPC remains stable. The location hierarchy was maintained in both years. TPC correlated most strongly with DPPH (*r* = 0.843, *p* < 0.001) and FRAP (*r* = 0.795); rosmarinic acid (Year 1) correlated with FRAP (*r* = 0.808) and DPPH (*r* = 0.685). Year-specific data are provided in Online Resource 1 (Tables S3–S6).

**Table 4:** Total phenolic content (TPC, mg GAE g⁻¹ DW), ABTS, DPPH, and FRAP (µmol TE g⁻¹ DW) of twelve *Ocimum basilicum* L. genotypes at three Turkish ecological sites (two-year means)

Genotype	TPC (mg GAE g⁻¹ DW)			ABTS (µmol TE g⁻¹ DW)			DPPH (µmol TE g⁻¹ DW)			FRAP (µmol TE g⁻¹ DW)		
	B	E	T	B	E	T	B	E	T	B	E	T
R-1	7.6 ^*c*^	12.2 ^*def*^	9.4 ^*c*^	232 ^*bc*^	423 ^*cd*^	307 ^*g*^	93 ^*fg*^	174 ^*d*^	125 ^*g*^	123 ^*ef*^	185 ^*ef*^	162 ^*g*^
R-3k	7.7 ^*c*^	13.2 ^*cde*^	18.0 ^*a*^	203 ^*cd*^	386 ^*de*^	594 ^*a*^	101 ^*ef*^	170 ^*de*^	339 ^*bc*^	141 ^*d*^	200 ^*cde*^	320 ^*ab*^
R-4	9.4 ^*b*^	10.6 ^*gh*^	16.6 ^*a*^	238 ^*b*^	326 ^*g*^	512 ^*cd*^	114 ^*bcd*^	134 ^*f*^	261 ^*ef*^	149 ^*cd*^	166 ^*fg*^	300 ^*bc*^
R-10 A	9.4 ^*b*^	16.8 ^*a*^	17.9 ^*a*^	279 ^*a*^	496 ^*ab*^	533 ^*bcd*^	135 ^*a*^	360 ^*a*^	343 ^*b*^	179 ^*ab*^	293 ^*a*^	288 ^*bcd*^
R-15	7.5 ^*cd*^	11.1 ^*fgh*^	8.6 ^*c*^	188 ^*de*^	333 ^*fg*^	378 ^*f*^	80 ^*h*^	161 ^*de*^	239 ^*f*^	120 ^*ef*^	211 ^*cd*^	248 ^*ef*^
R-16	9.3 ^*b*^	13.6 ^*bcd*^	16.9 ^*a*^	173 ^*e*^	415 ^*de*^	482 ^*de*^	60 ^*i*^	147 ^*ef*^	233 ^*f*^	98 ^*g*^	187 ^*def*^	191 ^*g*^
R-17	7.0 ^*cd*^	14.8 ^*b*^	16.9 ^*a*^	240 ^*b*^	419 ^*d*^	575 ^*ab*^	107 ^*cde*^	280 ^*b*^	357 ^*ab*^	137 ^*de*^	244 ^*b*^	281 ^*cde*^
R-19	7.1 ^*cd*^	9.7 ^*h*^	13.7 ^*b*^	237 ^*b*^	318 ^*g*^	389 ^*f*^	103 ^*def*^	170 ^*de*^	392 ^*a*^	165 ^*bc*^	215 ^*c*^	336 ^*a*^
R-20	6.4 ^*d*^	14.1 ^*bc*^	13.1 ^*b*^	237 ^*b*^	373 ^*ef*^	450 ^*e*^	51 ^*i*^	104 ^*g*^	228 ^*f*^	72 ^*h*^	149 ^*g*^	230 ^*f*^
R-23	11.2 ^*a*^	14.0 ^*bc*^	17.0 ^*a*^	239 ^*b*^	525 ^*a*^	558 ^*abc*^	119 ^*b*^	176 ^*d*^	307 ^*cd*^	155 ^*cd*^	212 ^*c*^	263 ^*def*^
Y-7	7.1 ^*cd*^	13.9 ^*bc*^	17.8 ^*a*^	259 ^*ab*^	322 ^*g*^	535 ^*bcd*^	115 ^*bc*^	230 ^*c*^	292 ^*de*^	193 ^*a*^	246 ^*b*^	263 ^*def*^
Y-15	7.5 ^*cd*^	12.0 ^*efg*^	18.6 ^*a*^	194 ^*de*^	468 ^*bc*^	486 ^*de*^	88 ^*gh*^	173 ^*de*^	370 ^*ab*^	115 ^*fg*^	197 ^*cde*^	306 ^*abc*^
*Location mean — Bursa*	8.1	–	–	227	–	–	97	–	–	137	–	–
*Location mean — Eskişehir*	–	13.0	–	–	400	–	–	190	–	–	209	–
Location mean — Tokat	–	–	15.4	–	–	483	–	–	291	–	–	266

Two-year means (*n* = 6 plots per genotype–location). B = Bursa; E = Eskişehir; T = Tokat. Location means (highlighted rows) are the 12-genotype average for each site. Within each location column, means followed by the same superscript letter do not differ significantly (Tukey’s HSD, α = 0.05). Compact-letter displays were generated from Tukey’s HSD post hoc comparisons of the corresponding SAS PROC GLM model. Year-specific means ± SD in Online Resource 1 (Tables S3–S6); three-way ANOVA summary in Table S12

#### Principal Component Analysis

**Fig. 3:**
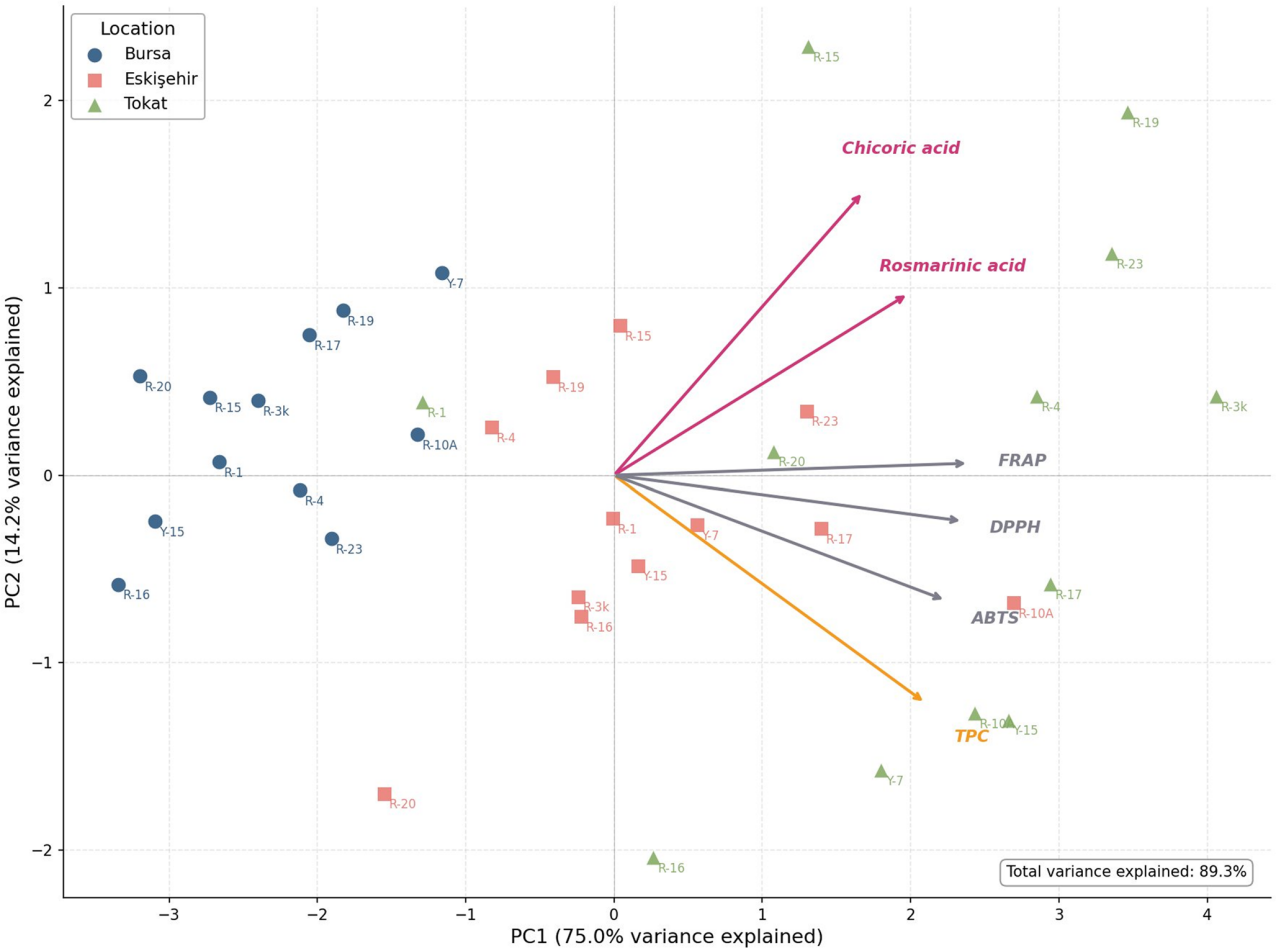
PCA biplot of six phenolic and antioxidant quality variables for twelve basil genotypes at three Turkish ecological sites (*n* = 36 genotype–location combinations). Rosmarinic acid and chicoric acid values shown are Year 1 data (HPLC–TOF); TPC, ABTS, DPPH, and FRAP values shown are two-year means (Year 1 + Year 2). PC1 = 75.0%; PC2 = 14.2%. Circles, Bursa; squares, Eskişehir; triangles, Tokat. Loading vectors indicate the direction and magnitude of variable contributions

PCA of the six quality variables explained 89.3% of the total variance in two components (PC1 = 75.0%; PC2 = 14.2%; Fig. 3). PC1 separated genotype–location combinations along a Tokat–Bursa quality axis. All loading vectors were oriented positively, and the Tokat combinations had the highest mean PC1 score (Tokat = + 2.05; Bursa = − 2.29), indicating consistently higher values across the six quality variables. Some genotype effects were still evident within less favourable sites: R-10 A and R-17 at Eskişehir had TPC values of 16.8 and 14.8 mg GAE g⁻¹ DW, respectively, within the Tokat range (8.6–18.6), and R-23 at Eskişehir had an ABTS value (525 µmol TE g⁻¹ DW) higher than several Tokat genotype means. Thus, favourable genotypes can partly offset site limitations. PC2 contrasted chicoric acid (loading = + 0.663) with TPC (− 0.532), pointing to differences between compound-specific and global phenolic measurements. Climate variables were not included in the PCA because meteorological data are constant within each site and do not vary across genotypes (*n* = 3 site values replicated across 12 genotypes = 36 observations); including them would create pseudo-replication and mix site-level with genotypic sources of variance. PCA loading values are provided in Online Resource 1 (Table S7).

## Conclusions

Location and genotype strongly influenced the individual phenolic profile in Year 1, while TPC and antioxidant capacity showed a stable location ranking across two growing seasons at three Turkish sites. Tokat ranked highest for individual phenolic concentrations (Year 1 HPLC–TOF data) and for two-year mean antioxidant capacity across all three assays. The location hierarchy remained stable across years for TPC, whereas antioxidant capacity increased substantially in Year 2, corresponding to higher precipitation. Based on Year 1 individual phenolic profiles and two-year antioxidant capacity data, genotypes R-23 and R-4 emerged as strong candidates for rosmarinic- and chicoric-acid-targeted cultivation at high-performing sites, while Y-15 exhibited the highest total phenolic content at Tokat. At Bursa, developmental-stage effects were genotype-dependent. Harvesting at the vegetative stage generally favoured rosmarinic acid accumulation, while harvesting at FF maximised TPC in high-accumulating genotypes (Y-7, R-17), consistent with a stage-dependent shift in the relative contribution of rosmarinic acid to the Folin-reactive phenolic pool. These patterns reflect site-specific ontogenetic dynamics at Bursa only; therefore, multi-location stage trials and postharvest evaluation are required before stage-specific harvest recommendations can be applied operationally. Several limitations should be noted. First, individual phenolic profiles are based solely on Year 1 HPLC–TOF data, as Year 2 data did not pass pre-submission quality control. Second, developmental-stage effects were characterised at a single site (Bursa, Year 1) and may not be applicable to other environments. Third, the study did not measure biosynthetic pathway intermediates or enzyme activities; thus, mechanistic interpretations remain correlative. Finally, postharvest stability and processing effects on phenolic content were not evaluated.

## Supplementary Information

Below is the link to the electronic supplementary material.

Supplementary Material 1

## Data Availability

Data are available from the corresponding authors upon reasonable request.

## Abbreviations

ABTS: 2,2′-Azinobis(3-ethylbenzothiazoline-6-sulphonic acid)
ANOVA: Analysis of Variance
CA: Chicoric Acid
DPPH: 2,2-Diphenyl-1-Picrylhydrazyl
DW: Dry Weight
FF: Full Flowering
FHY: Fresh Herb Yield
FO: Flowering Onset
FRAP: Ferric Reducing Antioxidant Power
G×L: Genotype × Location Interaction
GAE: Gallic Acid Equivalent
GS: Growing Season
HPLC–TOF: High-Performance Liquid Chromatography-Time-of-Flight
LOD: Limit of Detection
LOQ: Limit of Quantification
OM: Organic Matter
PAL: Phenylalanine Ammonia-Lyase
PCA: Principal Component Analysis
RA: Rosmarinic Acid
RCBD: Randomised Complete Block Design
RH: Relative Humidity
SS: Sum of Squares
TE: Trolox Equivalent
TPC: Total Phenolic Content
V: Vegetative Stage
