# Supplementary material for: Phenolics and Antioxidant Capacity of Basil (Ocimum basilicum L.) Genotypes Across Locations and Developmental Stages
Source: Plant Foods Hum Nutr. 2026 May 13;81(2):57. doi: 10.1007/s11130-026-01512-1 (PMC13171659; doi:10.1007/s11130-026-01512-1)

**Supplementary Material**

Phenolics and Antioxidant Capacity of Basil (*Ocimum basilicum* L.) Genotypes Across Locations and Developmental Stages

Plant Foods for Human Nutrition

Adem Zorlu, İsa Telci, Mahfuz Elmastaş, Oya Kaçar, Zehra Aytaç, Nusret Genç, Ömer Kayır

Corresponding authors: Adem Zorlu, Department of Phytotherapy, Hamidiye Institute of Health Sciences, University of Health Sciences, Üsküdar, Istanbul, Türkiye (drademzorlu@gmail.com); İsa Telci, Department of Industrial Crops, Isparta University of Applied Sciences, Isparta, Türkiye (isatelci@isparta.edu.tr).

This supplementary file contains Tables S1–S15 and Figs. S1–S2. Tables S1–S2 provide genotype characterisation and Year 1 nine-compound HPLC–TOF phenolic profiles. Tables S3–S6 give year-wise TPC, ABTS, DPPH, and FRAP data; Table S7. gives PCA loadings; Table S8. gives HPLC–TOF validation parameters; Tables S9–S11 summarise the Bursa Year 1 developmental-stage experiment; and Tables S12–S15 provide ANOVA summaries. Fig. S1. shows plant height and fresh herb yield, and Fig. S2. shows Year 1 rosmarinic acid and chicoric acid by location.

**Table S1.** Characterisation of the twelve *Ocimum basilicum* L. genotypes.

| Code | Type | Origin region | Collection year | Leaf colour | Chemotype | Selection notes |
| --- | --- | --- | --- | --- | --- | --- |
| R-1 | Landrace | Artvin | 2006 | Green | RA-rich | Selected for phenolic quality diversity |
| R-3k | Landrace | Samsun | 2005 | Green | RA-rich | High RA at Tokat 2012 screening |
| R-4 | Landrace | Trabzon | 2007 | Green | RA-rich | High yield + RA combination |
| R-10A | Landrace | Kastamonu | 2004 | Green | RA-rich | Maximum plant height + yield |
| R-15 | Landrace | Tokat | 2003 | Green | RA-mid | Local reference genotype |
| R-16 | Landrace | Rize | 2008 | Green | Low-phenolic | Low RA and CA reference at all locations |
| R-17 | Landrace | Giresun | 2006 | Green | RA-rich | High FHY + RA combination |
| R-19 | Landrace | Samsun | 2005 | Green | RA+CA-rich | Dual RA+CA high accumulator |
| R-20 | Landrace | Ordu | 2007 | Green | RA-mid | High ABTS at Bursa |
| R-23 | Landrace | Zonguldak | 2010 | Green | CA-rich | Maximum CA at Eskişehir and Tokat; among top-three CA at Bursa |
| Y-7 | Cultivar (introduced) | Commercial source | — | Green | TPC-rich | High TPC accumulator at full flowering (Bursa stage); introduced cultivar |
| Y-15 | Cultivar (introduced) | Commercial source | — | Green | RA-mid | Introduced cultivar; broad-base comparison |
| Landraces: farmer-selected populations from multiple Turkish provinces (collected 2003–2010). Cultivars (Y-coded): commercial introductions. Chemotype classification based on HPLC–TOF Year 1 data and 2012 Tokat screening. | | | | | | |

**Table S2.** Individual HPLC–TOF phenolic concentrations (mg 100 g⁻¹ DW, mean ± SD, n = 3), Year 1.

| Compound | Location | R-1 | R-3k | R-4 | R-10A | R-15 | R-16 | R-17 | R-19 | R-20 | R-23 | Y-7 | Y-15 |
| --- | --- | --- | --- | --- | --- | --- | --- | --- | --- | --- | --- | --- | --- |
| 4-OH-Benzoic acid | Bursa | 0.15 ± 0.02 | 0.17 ± 0.02 | 0.18 ± 0.02 | 0.15 ± 0.02 | 0.26 ± 0.02 | 0.12 ± 0.02 | 0.11 ± 0.02 | 0.19 ± 0.01 | 0.13 ± 0.02 | 0.17 ± 0.02 | 0.10 ± 0.02 | 0.11 ± 0.02 |
|  | Eskişehir | 0.19 ± 0.01 | 0.16 ± 0.02 | 0.23 ± 0.03 | 0.13 ± 0.02 | 0.14 ± 0.02 | 0.22 ± 0.03 | 0.11 ± 0.02 | 0.13 ± 0.02 | 0.06 ± 0.01 | 0.12 ± 0.02 | 0.11 ± 0.02 | 0.11 ± 0.02 |
|  | Tokat | 0.25 ± 0.03 | 0.19 ± 0.01 | 0.20 ± 0.01 | 0.11 ± 0.02 | 0.13 ± 0.02 | 0.10 ± 0.02 | 0.09 ± 0.02 | 0.24 ± 0.01 | 0.15 ± 0.02 | 0.10 ± 0.02 | 0.12 ± 0.02 | 0.11 ± 0.02 |
| Ferulic acid | Bursa | 0.07 ± 0.02 | 0.11 ± 0.02 | 0.04 ± 0.01 | 0.23 ± 0.01 | 0.26 ± 0.03 | 0.00 ± 0.01 | 0.45 ± 0.03 | 0.21 ± 0.03 | 0.19 ± 0.03 | 0.13 ± 0.02 | 0.21 ± 0.03 | 0.05 ± 0.01 |
|  | Eskişehir | 0.05 ± 0.01 | 0.07 ± 0.02 | 0.08 ± 0.02 | 0.25 ± 0.01 | 0.17 ± 0.02 | 0.00 ± 0.01 | 0.27 ± 0.03 | 0.00 ± 0.02 | 0.06 ± 0.01 | 0.01 ± 0.01 | 0.00 ± 0.01 | 0.16 ± 0.02 |
|  | Tokat | 0.00 ± 0.01 | 0.30 ± 0.04 | 0.12 ± 0.02 | 0.19 ± 0.01 | 0.13 ± 0.01 | 0.00 ± 0.02 | 0.39 ± 0.04 | 0.07 ± 0.02 | 0.04 ± 0.01 | 0.09 ± 0.02 | 0.01 ± 0.01 | 0.04 ± 0.01 |
| Gallic acid | Bursa | 0.04 ± 0.01 | 0.06 ± 0.01 | 0.06 ± 0.01 | 0.05 ± 0.01 | 0.06 ± 0.01 | 0.05 ± 0.01 | 0.06 ± 0.01 | 0.05 ± 0.01 | 0.06 ± 0.01 | 0.06 ± 0.01 | 0.05 ± 0.01 | 0.06 ± 0.01 |
|  | Eskişehir | 0.07 ± 0.02 | 0.05 ± 0.01 | 0.06 ± 0.01 | 0.05 ± 0.01 | 0.05 ± 0.01 | 0.06 ± 0.01 | 0.05 ± 0.01 | 0.06 ± 0.01 | 0.03 ± 0.01 | 0.05 ± 0.01 | 0.06 ± 0.01 | 0.06 ± 0.01 |
|  | Tokat | 0.06 ± 0.01 | 0.03 ± 0.01 | 0.05 ± 0.01 | 0.05 ± 0.01 | 0.06 ± 0.01 | 0.05 ± 0.01 | 0.05 ± 0.01 | 0.05 ± 0.01 | 0.05 ± 0.01 | 0.06 ± 0.01 | 0.05 ± 0.01 | 0.06 ± 0.01 |
| Gentisic acid | Bursa | 0.09 ± 0.02 | 0.04 ± 0.01 | 0.06 ± 0.01 | 0.08 ± 0.02 | 0.03 ± 0.01 | 0.04 ± 0.01 | 0.08 ± 0.02 | 0.04 ± 0.01 | 0.02 ± 0.01 | 0.06 ± 0.01 | 0.16 ± 0.02 | 0.18 ± 0.02 |
|  | Eskişehir | 0.09 ± 0.02 | 0.04 ± 0.01 | 0.06 ± 0.01 | 0.07 ± 0.02 | 0.06 ± 0.01 | 0.13 ± 0.02 | 0.05 ± 0.01 | 0.06 ± 0.01 | 0.04 ± 0.01 | 0.05 ± 0.01 | 0.09 ± 0.02 | 0.18 ± 0.02 |
|  | Tokat | 0.07 ± 0.02 | 0.12 ± 0.02 | 0.07 ± 0.02 | 0.04 ± 0.01 | 0.06 ± 0.01 | 0.05 ± 0.01 | 0.02 ± 0.01 | 0.03 ± 0.01 | 0.03 ± 0.01 | 0.07 ± 0.02 | 0.03 ± 0.01 | 0.10 ± 0.02 |
| Caffeic acid | Bursa | 0.60 ± 0.06 | 0.61 ± 0.04 | 0.65 ± 0.04 | 0.58 ± 0.06 | 0.74 ± 0.05 | 0.51 ± 0.05 | 0.54 ± 0.05 | 0.86 ± 0.08 | 0.65 ± 0.04 | 0.38 ± 0.04 | 0.73 ± 0.05 | 0.50 ± 0.03 |
|  | Eskişehir | 0.45 ± 0.03 | 0.40 ± 0.05 | 0.48 ± 0.03 | 0.33 ± 0.02 | 0.43 ± 0.05 | 0.47 ± 0.05 | 0.47 ± 0.05 | 0.70 ± 0.05 | 0.29 ± 0.03 | 0.54 ± 0.05 | 0.51 ± 0.03 | 0.54 ± 0.06 |
|  | Tokat | 0.35 ± 0.05 | 0.51 ± 0.06 | 0.39 ± 0.02 | 0.39 ± 0.02 | 0.30 ± 0.05 | 0.44 ± 0.03 | 0.26 ± 0.03 | 0.53 ± 0.06 | 0.30 ± 0.03 | 0.30 ± 0.03 | 0.34 ± 0.02 | 0.28 ± 0.03 |
| Chicoric acid | Bursa | 16.93 ± 0.75 | 19.75 ± 1.12 | 19.66 ± 1.31 | 20.05 ± 0.98 | 17.72 ± 1.08 | 7.89 ± 0.32 | 20.69 ± 0.72 | 23.45 ± 1.13 | 23.43 ± 1.52 | 21.87 ± 0.91 | 17.46 ± 0.77 | 9.00 ± 0.46 |
|  | Eskişehir | 18.88 ± 0.98 | 17.09 ± 0.84 | 19.18 ± 1.08 | 23.30 ± 0.82 | 29.83 ± 1.58 | 13.40 ± 0.94 | 23.61 ± 0.83 | 23.81 ± 1.13 | 7.35 ± 0.38 | 37.32 ± 2.40 | 15.97 ± 0.93 | 12.99 ± 0.96 |
|  | Tokat | 25.65 ± 0.90 | 48.04 ± 1.68 | 41.73 ± 1.93 | 26.67 ± 1.25 | 50.30 ± 1.41 | 13.00 ± 0.86 | 33.45 ± 1.61 | 59.59 ± 2.09 | 28.13 ± 1.44 | 68.58 ± 4.46 | 21.69 ± 1.34 | 22.08 ± 1.44 |
| Rosmarinic acid | Bursa | 78.25 ± 3.78 | 97.19 ± 4.38 | 77.43 ± 4.89 | 130.77 ± 3.92 | 102.82 ± 3.05 | 58.97 ± 2.74 | 139.59 ± 6.87 | 137.57 ± 7.57 | 72.61 ± 2.54 | 61.97 ± 2.17 | 213.82 ± 8.63 | 73.54 ± 4.08 |
|  | Eskişehir | 183.71 ± 10.10 | 143.51 ± 4.31 | 181.77 ± 10.00 | 226.11 ± 6.78 | 198.38 ± 4.59 | 168.67 ± 5.06 | 206.11 ± 6.18 | 169.96 ± 6.62 | 69.23 ± 2.42 | 205.03 ± 6.15 | 203.80 ± 8.32 | 198.73 ± 8.12 |
|  | Tokat | 125.49 ± 6.42 | 261.83 ± 7.85 | 240.16 ± 10.18 | 152.11 ± 5.82 | 242.50 ± 5.09 | 90.54 ± 3.45 | 198.79 ± 6.73 | 251.38 ± 11.05 | 202.35 ± 6.07 | 195.55 ± 9.14 | 135.85 ± 7.47 | 179.54 ± 6.40 |
| Quercetin | Bursa | 0.06 ± 0.01 | 0.06 ± 0.01 | 0.06 ± 0.01 | 0.07 ± 0.02 | 0.06 ± 0.01 | 0.06 ± 0.01 | 0.06 ± 0.01 | 0.06 ± 0.01 | 0.06 ± 0.01 | 0.06 ± 0.01 | 0.06 ± 0.01 | 0.06 ± 0.01 |
|  | Eskişehir | 0.06 ± 0.01 | 0.06 ± 0.01 | 0.06 ± 0.01 | 0.09 ± 0.02 | 0.06 ± 0.01 | 0.07 ± 0.02 | 0.07 ± 0.02 | 0.06 ± 0.01 | 0.03 ± 0.01 | 0.06 ± 0.01 | 0.06 ± 0.01 | 0.06 ± 0.01 |
|  | Tokat | 0.06 ± 0.01 | 0.06 ± 0.01 | 0.06 ± 0.01 | 0.11 ± 0.02 | 0.06 ± 0.01 | 0.07 ± 0.02 | 0.07 ± 0.02 | 0.07 ± 0.02 | 0.06 ± 0.01 | 0.06 ± 0.01 | 0.06 ± 0.01 | 0.06 ± 0.01 |
| Rutin | Bursa | 0.05 ± 0.01 | 2.68 ± 0.14 | 2.42 ± 0.16 | 9.17 ± 0.51 | 4.09 ± 0.29 | 2.82 ± 0.15 | 1.03 ± 0.06 | 1.20 ± 0.09 | 5.49 ± 0.34 | 8.70 ± 0.61 | 7.36 ± 0.52 | 2.84 ± 0.22 |
|  | Eskişehir | 0.01 ± 0.01 | 6.88 ± 0.35 | 12.18 ± 0.65 | 6.17 ± 0.30 | 6.69 ± 0.53 | 11.97 ± 0.48 | 2.82 ± 0.25 | 6.86 ± 0.43 | 3.67 ± 0.22 | 25.13 ± 1.63 | 11.59 ± 0.66 | 12.43 ± 0.75 |
|  | Tokat | 0.08 ± 0.02 | 7.51 ± 0.56 | 37.37 ± 1.38 | 19.32 ± 1.54 | 30.46 ± 0.23 | 5.43 ± 0.36 | 6.27 ± 0.41 | 0.06 ± 0.01 | 27.54 ± 1.08 | 39.58 ± 1.87 | 23.08 ± 1.50 | 34.89 ± 2.25 |
| Mean ± SD (n = 3). Year 1. DW = dry weight. | | | | | | | | | | | | | |

Note for Table S2: Values reported as 0.00 indicate concentrations below the quantification limit after rounding and were not interpreted biologically.

**Table S3.** TPC (mg GAE g⁻¹ DW, mean ± SD, n = 3) by genotype, location, and year.

| Year / Location | R-1 | R-3k | R-4 | R-10A | R-15 | R-16 | R-17 | R-19 | R-20 | R-23 | Y-7 | Y-15 |
| --- | --- | --- | --- | --- | --- | --- | --- | --- | --- | --- | --- | --- |
| Year 1 — Bursa | 9.02 ± 0.93 | 8.44 ± 0.27 | 10.41 ± 0.70 | 8.18 ± 0.37 | 7.62 ± 0.33 | 14.08 ± 1.02 | 9.24 ± 0.45 | 8.13 ± 0.57 | 9.74 ± 0.31 | 12.65 ± 0.72 | 4.98 ± 0.25 | 9.23 ± 0.60 |
| Year 1 — Eskişehir | 10.42 ± 1.21 | 11.94 ± 0.63 | 11.93 ± 0.89 | 17.13 ± 0.55 | 12.74 ± 0.70 | 13.43 ± 0.98 | 13.47 ± 0.90 | 6.39 ± 0.28 | 19.43 ± 1.04 | 13.28 ± 0.74 | 10.02 ± 0.61 | 9.94 ± 0.70 |
| Year 1 — Tokat | 10.69 ± 1.21 | 16.51 ± 1.08 | 18.33 ± 1.42 | 18.46 ± 0.86 | 7.32 ± 0.40 | 17.58 ± 0.63 | 16.29 ± 0.76 | 7.19 ± 0.33 | 12.74 ± 0.56 | 18.78 ± 1.30 | 16.94 ± 0.73 | 19.50 ± 1.78 |
|  | | | | | | | | | | | | |
| Year 2 — Bursa | 6.10 ± 0.51 | 7.04 ± 0.27 | 8.43 ± 0.57 | 10.53 ± 0.72 | 7.38 ± 0.50 | 4.49 ± 0.11 | 4.69 ± 0.39 | 6.02 ± 0.38 | 3.13 ± 0.39 | 9.81 ± 1.02 | 9.13 ± 0.58 | 5.71 ± 0.37 |
| Year 2 — Eskişehir | 14.07 ± 0.82 | 14.45 ± 0.30 | 9.23 ± 0.51 | 16.37 ± 0.73 | 9.40 ± 0.23 | 13.70 ± 0.55 | 16.13 ± 0.87 | 13.08 ± 0.76 | 8.74 ± 0.09 | 14.81 ± 0.60 | 17.86 ± 1.10 | 14.15 ± 0.94 |
| Year 2 — Tokat | 8.18 ± 0.99 | 19.48 ± 0.88 | 14.96 ± 1.24 | 17.27 ± 0.68 | 9.95 ± 0.77 | 16.20 ± 0.82 | 17.59 ± 1.21 | 20.21 ± 1.33 | 13.41 ± 0.52 | 15.27 ± 0.99 | 18.66 ± 0.66 | 17.65 ± 0.70 |
|  | | | | | | | | | | | | |
| Two-year mean — Bursa | 7.6 | 7.7 | 9.4 | 9.4 | 7.5 | 9.3 | 7.0 | 7.1 | 6.4 | 11.2 | 7.1 | 7.5 |
| Two-year mean — Eskişehir | 12.2 | 13.2 | 10.6 | 16.8 | 11.1 | 13.6 | 14.8 | 9.7 | 14.1 | 14.0 | 13.9 | 12.0 |
| Two-year mean — Tokat | 9.4 | 18.0 | 16.6 | 17.9 | 8.6 | 16.9 | 16.9 | 13.7 | 13.1 | 17.0 | 17.8 | 18.6 |
| TPC in mg GAE g⁻¹ DW. n = 3 RCBD plot replicates. Two-year mean calculated from the displayed Year 1 and Year 2 means using round-half-up to the reported precision (one decimal place). | | | | | | | | | | | | |

**Table S4.** ABTS (µmol TE g⁻¹ DW, mean ± SD, n = 3) by genotype, location, and year.

| Year / Location | R-1 | R-3k | R-4 | R-10A | R-15 | R-16 | R-17 | R-19 | R-20 | R-23 | Y-7 | Y-15 |
| --- | --- | --- | --- | --- | --- | --- | --- | --- | --- | --- | --- | --- |
| Year 1 — Bursa | 253 ± 12 | 221 ± 7 | 191 ± 10 | 270 ± 11 | 206 ± 7 | 204 ± 13 | 325 ± 10 | 366 ± 30 | 176 ± 6 | 223 ± 15 | 365 ± 20 | 255 ± 16 |
| Year 1 — Eskişehir | 298 ± 17 | 297 ± 13 | 303 ± 18 | 415 ± 13 | 385 ± 18 | 331 ± 20 | 336 ± 12 | 382 ± 20 | 262 ± 10 | 338 ± 23 | 334 ± 21 | 350 ± 19 |
| Year 1 — Tokat | 320 ± 17 | 381 ± 22 | 379 ± 25 | 310 ± 13 | 383 ± 18 | 223 ± 8 | 389 ± 16 | 387 ± 19 | 384 ± 16 | 389 ± 32 | 362 ± 16 | 384 ± 28 |
|  | | | | | | | | | | | | |
| Year 2 — Bursa | 211 ± 7 | 185 ± 8 | 285 ± 16 | 288 ± 12 | 170 ± 11 | 141 ± 8 | 155 ± 12 | 107 ± 5 | 298 ± 24 | 254 ± 27 | 152 ± 10 | 132 ± 18 |
| Year 2 — Eskişehir | 548 ± 19 | 475 ± 22 | 348 ± 16 | 576 ± 35 | 281 ± 12 | 498 ± 29 | 502 ± 24 | 253 ± 14 | 483 ± 23 | 712 ± 39 | 309 ± 20 | 586 ± 50 |
| Year 2 — Tokat | 293 ± 19 | 807 ± 42 | 645 ± 48 | 756 ± 36 | 372 ± 22 | 741 ± 36 | 760 ± 42 | 390 ± 16 | 515 ± 29 | 727 ± 57 | 707 ± 29 | 588 ± 33 |
|  | | | | | | | | | | | | |
| Two-year mean — Bursa | 232 | 203 | 238 | 279 | 188 | 173 | 240 | 237 | 237 | 239 | 259 | 194 |
| Two-year mean — Eskişehir | 423 | 386 | 326 | 496 | 333 | 415 | 419 | 318 | 373 | 525 | 322 | 468 |
| Two-year mean — Tokat | 307 | 594 | 512 | 533 | 378 | 482 | 575 | 389 | 450 | 558 | 535 | 486 |
| ABTS in µmol TE g⁻¹ DW. n = 3 RCBD plot replicates. Two-year mean calculated from the displayed Year 1 and Year 2 means using round-half-up to the reported precision (nearest integer). | | | | | | | | | | | | |

**Table S5.** DPPH (µmol TE g⁻¹ DW, mean ± SD, n = 3) by genotype, location, and year.

| Year / Location | R-1 | R-3k | R-4 | R-10A | R-15 | R-16 | R-17 | R-19 | R-20 | R-23 | Y-7 | Y-15 |
| --- | --- | --- | --- | --- | --- | --- | --- | --- | --- | --- | --- | --- |
| Year 1 — Bursa | 68 ± 4 | 91 ± 4 | 77 ± 3 | 53 ± 3 | 64 ± 2 | 62 ± 4 | 140 ± 5 | 116 ± 8 | 36 ± 2 | 82 ± 5 | 86 ± 5 | 82 ± 4 |
| Year 1 — Eskişehir | 90 ± 6 | 100 ± 6 | 101 ± 5 | 273 ± 10 | 176 ± 8 | 90 ± 6 | 148 ± 8 | 115 ± 5 | 82 ± 5 | 109 ± 6 | 101 ± 7 | 93 ± 5 |
| Year 1 — Tokat | 116 ± 8 | 223 ± 17 | 209 ± 13 | 165 ± 8 | 286 ± 14 | 58 ± 2 | 276 ± 9 | 280 ± 12 | 210 ± 9 | 254 ± 19 | 161 ± 8 | 338 ± 24 |
|  | | | | | | | | | | | | |
| Year 2 — Bursa | 118 ± 5 | 110 ± 5 | 151 ± 7 | 217 ± 12 | 95 ± 4 | 58 ± 3 | 74 ± 3 | 90 ± 4 | 65 ± 4 | 156 ± 11 | 143 ± 10 | 93 ± 5 |
| Year 2 — Eskişehir | 258 ± 12 | 239 ± 13 | 167 ± 6 | 447 ± 31 | 145 ± 6 | 204 ± 11 | 411 ± 27 | 225 ± 11 | 126 ± 7 | 242 ± 10 | 358 ± 28 | 253 ± 21 |
| Year 2 — Tokat | 134 ± 10 | 455 ± 32 | 312 ± 21 | 521 ± 29 | 191 ± 11 | 407 ± 17 | 437 ± 26 | 504 ± 27 | 245 ± 8 | 359 ± 25 | 422 ± 18 | 402 ± 20 |
|  | | | | | | | | | | | | |
| Two-year mean — Bursa | 93 | 101 | 114 | 135 | 80 | 60 | 107 | 103 | 51 | 119 | 115 | 88 |
| Two-year mean — Eskişehir | 174 | 170 | 134 | 360 | 161 | 147 | 280 | 170 | 104 | 176 | 230 | 173 |
| Two-year mean — Tokat | 125 | 339 | 261 | 343 | 239 | 233 | 357 | 392 | 228 | 307 | 292 | 370 |
| DPPH in µmol TE g⁻¹ DW. n = 3 RCBD plot replicates. Two-year mean calculated from the displayed Year 1 and Year 2 means using round-half-up to the reported precision (nearest integer). | | | | | | | | | | | | |

**Table S6.** FRAP (µmol TE g⁻¹ DW, mean ± SD, n = 3) by genotype, location, and year.

| Year / Location | R-1 | R-3k | R-4 | R-10A | R-15 | R-16 | R-17 | R-19 | R-20 | R-23 | Y-7 | Y-15 |
| --- | --- | --- | --- | --- | --- | --- | --- | --- | --- | --- | --- | --- |
| Year 1 — Bursa | 133 ± 7 | 148 ± 5 | 126 ± 12 | 165 ± 7 | 124 ± 6 | 116 ± 7 | 197 ± 10 | 224 ± 19 | 86 ± 3 | 131 ± 8 | 234 ± 10 | 134 ± 10 |
| Year 1 — Eskişehir | 168 ± 10 | 183 ± 9 | 174 ± 19 | 322 ± 11 | 268 ± 16 | 180 ± 11 | 207 ± 13 | 210 ± 11 | 170 ± 11 | 186 ± 11 | 205 ± 11 | 178 ± 9 |
| Year 1 — Tokat | 191 ± 11 | 337 ± 21 | 329 ± 39 | 230 ± 10 | 326 ± 20 | 108 ± 4 | 286 ± 13 | 326 ± 20 | 263 ± 14 | 287 ± 22 | 219 ± 9 | 339 ± 23 |
|  | | | | | | | | | | | | |
| Year 2 — Bursa | 113 ± 4 | 134 ± 6 | 172 ± 18 | 192 ± 9 | 116 ± 8 | 80 ± 4 | 77 ± 4 | 106 ± 6 | 58 ± 4 | 178 ± 13 | 152 ± 9 | 96 ± 5 |
| Year 2 — Eskişehir | 202 ± 8 | 217 ± 10 | 158 ± 14 | 263 ± 15 | 153 ± 9 | 193 ± 11 | 281 ± 20 | 220 ± 14 | 128 ± 8 | 238 ± 10 | 286 ± 17 | 215 ± 16 |
| Year 2 — Tokat | 132 ± 9 | 302 ± 16 | 271 ± 32 | 345 ± 16 | 169 ± 12 | 274 ± 11 | 275 ± 17 | 346 ± 21 | 196 ± 10 | 239 ± 17 | 307 ± 10 | 273 ± 15 |
|  | | | | | | | | | | | | |
| Two-year mean — Bursa | 123 | 141 | 149 | 179 | 120 | 98 | 137 | 165 | 72 | 155 | 193 | 115 |
| Two-year mean — Eskişehir | 185 | 200 | 166 | 293 | 211 | 187 | 244 | 215 | 149 | 212 | 246 | 197 |
| Two-year mean — Tokat | 162 | 320 | 300 | 288 | 248 | 191 | 281 | 336 | 230 | 263 | 263 | 306 |
| FRAP in µmol TE g⁻¹ DW. n = 3 RCBD plot replicates. Two-year mean calculated from the displayed Year 1 and Year 2 means using round-half-up to the reported precision (nearest integer). | | | | | | | | | | | | |

**Table S7.** PCA component loadings (n = 36 genotype–location combinations).

| Variable | PC1 loading | PC2 loading |
| --- | --- | --- |
| Rosmarinic acid (Year 1) | +0.3790 | +0.4245 |
| Chicoric acid (Year 1) | +0.3208 | +0.6626 |
| TPC (two-year mean) | +0.4008 | -0.5322 |
| ABTS (two-year mean) | +0.4272 | -0.2922 |
| DPPH (two-year mean) | +0.4492 | -0.1068 |
| FRAP (two-year mean) | +0.4567 | +0.0279 |
| Eigenvalue | 4.5018 | 0.8550 |
| Variance explained (%) | 75.03% | 14.25% |
| Cumulative variance (%) | 75.03% | 89.28% |
| PCA on z-scored data (n = 36). | | |

**Table S8.** HPLC–TOF method validation parameters for the nine quantified phenolic compounds.

| Compound | RT (min) | Identification ion (m/z) | Calibration equation | R² | LOD (µg L⁻¹) | LOQ (µg L⁻¹) | Range (ppb) |
| --- | --- | --- | --- | --- | --- | --- | --- |
| Gallic acid | 2.552 | [M−H]⁻ 169.0112 | y = 631.2x − 3028.9 | 0.985 | 3.52 | 10.67 | 25–2500 |
| Gentisic acid | 4.251 | [M−H]⁻ 153.0163 | y = 577.1x − 418.6 | 0.995 | 3.57 | 10.80 | 25–2500 |
| 4-OH-Benzoic acid | 6.671 | [M−H]⁻ 137.0217 | y = 403.9x − 3177.8 | 0.997 | 3.71 | 11.24 | 25–2500 |
| Caffeic acid | 7.632 | [M−H]⁻ 179.0316 | y = 1275.4x + 8066.5 | 0.985 | 2.98 | 9.03 | 25–2500 |
| Rutin | 9.908 | [M−H]⁻ 609.1395 | y = 983.5x + 2546.0 | 0.992 | 3.23 | 9.77 | 25–2500 |
| Chicoric acid | 10.020 | [M−H]⁻ 473.0731 | y = 812.6x + 18220.0 | 0.987 | 3.37 | 10.20 | 25–2500 |
| Ferulic acid | 10.934 | [M−H]⁻ 193.0469 | y = 330.0x + 6138.5 | 0.988 | 3.77 | 11.42 | 25–2500 |
| Rosmarinic acid | 12.536 | [M−H]⁻ 359.0732 | y = 799.7x + 6342.2 | 0.985 | 3.38 | 10.24 | 25–2500 |
| Quercetin | 15.485 | [M−H]⁻ 301.0317 | y = 2659.8x − 15345.0 | 0.989 | 1.83 | 5.55 | 25–2500 |
| Instrument: Agilent 1200 Series HPLC coupled to Bruker MicrOTOF mass spectrometer. Column: ZORBAX SB-C18 (150 × 4.6 mm, 3.5 µm). Identification in negative ionisation mode [M−H]⁻. LOD calculated at S/N = 3; LOQ at S/N = 10. Calibration range: seven concentration levels (25–2500 ppb). Extracts were screened against a 22-compound reference standard panel; nine compounds were detected and quantified in basil samples. RT = retention time; LOD = limit of detection; LOQ = limit of quantification; ppb = µg L⁻¹ = ng mL⁻¹. Standards: Sigma-Aldrich, Steinheim, Germany (purity ≥ 98%). | | | | | | | |

**Table S9.** Individual HPLC–TOF phenolic compound concentrations (mg 100 g⁻¹ DW) of twelve *Ocimum basilicum* L. genotypes at three developmental stages; Bursa site, Year 1.

| **Compound** | **R-1** | | | **R-3k** | | | **R-4** | | | **R-10A** | | | **R-15** | | | **R-16** | | | | **R-17** | | | | **R-19** | | | | **R-20** | | | | **R-23** | | | | **Y-7** | | | | **Y-15** | | | |  |
| --- | --- | --- | --- | --- | --- | --- | --- | --- | --- | --- | --- | --- | --- | --- | --- | --- | --- | --- | --- | --- | --- | --- | --- | --- | --- | --- | --- | --- | --- | --- | --- | --- | --- | --- | --- | --- | --- | --- | --- | --- | --- | --- | --- | --- |
|  | **V** | **FO** | **FF** | **V** | **FO** | **FF** | **V** | **FO** | **FF** | **V** | **FO** | **FF** | **V** | **FO** | **FF** | **V** | **FO** | **FF** | **V** | | **FO** | **FF** | **V** | | **FO** | **FF** | **V** | | **FO** | **FF** | **V** | | **FO** | **FF** | **V** | | **FO** | **FF** | **V** | | **FO** | **FF** |  |  |
| *4-OH-Benzoic acid* | 0.16 | 0.28 | 0.15 | 0.12 | 0.32 | 0.16 | 0.19 | 0.14 | 0.10 | 0.30 | 0.15 | 0.12 | 0.26 | 0.10 | 0.13 | 0.14 | 0.30 | 0.33 | 0.09 | | 0.10 | 0.13 | 0.27 | | 0.11 | 0.32 | 0.26 | | 0.42 | 0.14 | 0.12 | | 0.19 | 0.16 | 0.10 | | 0.13 | 0.28 | 0.25 | | 0.11 | 0.20 |  |  |
| *Ferulic acid* | 0.30 | 0.17 | 0.07 | 0.24 | 0.31 | 0.11 | 0.04 | 0.00 | 0.02 | 0.00 | 0.24 | 0.47 | 0.25 | 0.14 | 0.11 | 0.01 | 0.11 | 0.26 | 0.00 | | 0.13 | 0.05 | 0.03 | | 0.13 | 0.35 | 0.10 | | 0.13 | 0.28 | 0.00 | | 0.21 | 0.13 | 0.00 | | 0.35 | 0.28 | 0.05 | | 0.05 | 0.07 |  |  |
| *Gallic acid* | 0.06 | 0.06 | 0.05 | 0.04 | 0.06 | 0.06 | 0.06 | 0.05 | 0.05 | 0.05 | 0.06 | 0.05 | 0.06 | 0.06 | 0.06 | 0.05 | 0.05 | 0.06 | 0.06 | | 0.06 | 0.05 | 0.07 | | 0.05 | 0.06 | 0.06 | | 0.07 | 0.06 | 0.05 | | 0.06 | 0.06 | 0.05 | | 0.06 | 0.07 | 0.08 | | 0.06 | 0.07 |  |  |
| *Gentisic acid* | 0.08 | 0.15 | 0.09 | 0.07 | 0.07 | 0.04 | 0.06 | 0.06 | 0.07 | 0.06 | 0.08 | 0.08 | 0.03 | 0.07 | 0.08 | 0.12 | 0.08 | 0.06 | 0.05 | | 0.05 | 0.05 | 0.12 | | 0.10 | 0.12 | 0.04 | | 0.15 | 0.02 | 0.04 | | 0.04 | 0.06 | 0.07 | | 0.05 | 0.04 | 0.14 | | 0.18 | 0.17 |  |  |
| *Caffeic acid* | 0.95 | 0.90 | 0.61 | 0.57 | 1.1 | 0.60 | 0.66 | 0.40 | 0.39 | 0.47 | 0.57 | 0.53 | 0.72 | 0.47 | 0.48 | 0.75 | 0.61 | 0.75 | 0.37 | | 0.63 | 0.41 | 0.77 | | 0.72 | 0.85 | 0.75 | | 0.89 | 0.65 | 0.52 | | 0.88 | 0.39 | 0.31 | | 0.43 | 0.39 | 0.73 | | 0.50 | 0.37 |  |  |
| *Chicoric acid* | 20.6 | 17.1 | 12.6 | 16.2 | 19.7 | 18.6 | 19.6 | 19.6 | 10.3 | 29.2 | 19.5 | 18.4 | 20.6 | 17.7 | 14.9 | 10.1 | 7.9 | 9.7 | 16.7 | | 20.2 | 15.8 | 18.0 | | 23.8 | 19.0 | 23.2 | | 23.5 | 16.3 | 37.6 | | 22.1 | 17.4 | 18.8 | | 20.8 | 13.5 | 15.3 | | 8.9 | 8.7 |  |  |
| ***Rosmarinic acid*** | **178.6** | 80.1 | 58.1 | **97.2** | 97.7 | 70.6 | **134.8** | 77.8 | 59.4 | **118.8** | 130.8 | 138.2 | **148.7** | 102.8 | 94.6 | **103.7** | 59.0 | 53.8 | **136.9** | | 141.8 | 173.6 | **98.7** | | 136.6 | 82.8 | **79.8** | | 71.8 | 84.5 | **136.2** | | 63.2 | 71.3 | **152.8** | | 214.6 | 218.3 | **70.6** | | 72.3 | 62.3 |  |  |
| *Quercetin* | 0.06 | 0.06 | 0.06 | 0.06 | 0.06 | 0.06 | 0.06 | 0.07 | 0.06 | 0.06 | 0.06 | 0.06 | 0.06 | 0.06 | 0.06 | 0.07 | 0.08 | 0.07 | 0.06 | | 0.07 | 0.06 | 0.06 | | 0.06 | 0.06 | 0.06 | | 0.06 | 0.06 | 0.06 | | 0.06 | 0.06 | 0.06 | | 0.06 | 0.06 | 0.06 | | 0.06 | 0.06 |  |  |
| *Rutin* | 0.05 | 1.4 | 0.05 | 1.6 | 2.9 | 2.7 | 2.4 | 0.03 | 3.0 | 1.1 | 9.1 | 1.0 | 4.0 | 4.0 | 3.5 | 3.3 | 0.01 | 6.5 | 5.8 | | 1.6 | 10.2 | 1.3 | | 1.1 | 0.60 | 3.0 | | 4.8 | 5.4 | 2.8 | | 1.2 | 8.8 | 7.4 | | 8.7 | 20.6 | 1.4 | | 2.8 | 6.3 |  |  |
| *Bursa site, Year 1. Values are means of three replicate plots (n = 3). V = vegetative; FO = flowering onset; FF = full flowering. Units: mg 100 g⁻¹ DW. Source: TÜBİTAK project report (Table 4.39). Year 2 individual HPLC–TOF data are excluded following pre-submission quality control.* | | | | | | | | | | | | | | | | | | | | | | | | | | | | | | | | | | | | | | | | | | | | |

**Table S10.** Antioxidant capacity (µmol TE g⁻¹ DW) of twelve *Ocimum basilicum* L. genotypes at three developmental stages; Bursa site, Year 1. ABTS, DPPH, and FRAP assays.

| **Assay** | **R-1** | | | **R-3k** | | | **R-4** | | | **R-10A** | | | **R-15** | | | **R-16** | | | **R-17** | | | **R-19** | | | **R-20** | | | **R-23** | | | **Y-7** | | | **Y-15** | | |  |
| --- | --- | --- | --- | --- | --- | --- | --- | --- | --- | --- | --- | --- | --- | --- | --- | --- | --- | --- | --- | --- | --- | --- | --- | --- | --- | --- | --- | --- | --- | --- | --- | --- | --- | --- | --- | --- | --- |
|  | **V** | **FO** | **FF** | **V** | **FO** | **FF** | **V** | **FO** | **FF** | **V** | **FO** | **FF** | **V** | **FO** | **FF** | **V** | **FO** | **FF** | **V** | **FO** | **FF** | **V** | **FO** | **FF** | **V** | **FO** | **FF** | **V** | **FO** | **FF** | **V** | **FO** | **FF** | **V** | **FO** | **FF** |  |
| **ABTS (µmol TE g⁻¹ DW)** | 268.3 | 254.1 | 192.4 | 189.3 | 219.9 | 179.3 | 205.3 | 192.1 | 217.9 | 182.0 | 265.8 | 231.9 | 233.9 | 207.0 | 145.9 | 227.9 | 203.3 | 201.2 | 204.6 | 332.7 | 306.9 | 347.5 | 363.0 | 220.6 | 185.3 | 172.8 | 216.9 | 245.1 | 224.2 | 204.1 | 288.4 | 366.9 | 372.0 | 224.2 | 251.3 | 251.6 |  |
| **DPPH (µmol TE g⁻¹ DW)** | 72.0 | 67.7 | 49.2 | 39.8 | 91.4 | 60.2 | 43.8 | 78.3 | 77.6 | 43.0 | 53.1 | 77.4 | 64.6 | 63.5 | 49.1 | 65.4 | 61.9 | 69.0 | 60.0 | 142.4 | 117.7 | 88.4 | 115.6 | 59.9 | 51.0 | 36.3 | 65.8 | 63.4 | 81.7 | 57.1 | 88.9 | 86.5 | 182.8 | 48.3 | 80.5 | 85.6 |  |
| **FRAP (µmol TE g⁻¹ DW)** | 156.0 | 133.2 | 89.8 | 110.7 | 147.0 | 135.3 | 114.4 | 125.6 | 111.0 | 100.6 | 163.9 | 147.6 | 137.3 | 122.7 | 91.6 | 119.0 | 116.2 | 118.2 | 124.2 | 199.6 | 226.7 | 186.3 | 223.9 | 123.1 | 105.9 | 86.0 | 136.8 | 137.8 | 132.2 | 118.3 | 166.8 | 234.9 | 339.2 | 116.0 | 132.4 | 136.9 |  |
| *Bursa site, Year 1 only. This table reports the developmental-stage antioxidant dataset collected alongside the Bursa Year 1 stage experiment. The across-location antioxidant dataset for both years is reported separately in Tables S4–S6 and was not affected by the Year 2 HPLC–TOF exclusion. V = vegetative; FO = flowering onset; FF = full flowering. TE = Trolox equivalent. Source: TÜBİTAK project report (Tables 4.44–4.46).* | | | | | | | | | | | | | | | | | | | | | | | | | | | | | | | | | | | | | |

**Table S11.** Developmental stage means for rosmarinic acid (RA, mg 100 g⁻¹ DW), total phenolic content (TPC, mg GAE g⁻¹ DW), ABTS, DPPH and FRAP antioxidant capacity (µmol TE g⁻¹ DW) in twelve *Ocimum basilicum* L. genotypes; Bursa site, Year 1. V = vegetative; FO = flowering onset; FF = full flowering.

| **Assay / Stage** | **R-1** | | | **R-3k** | | | **R-4** | | | **R-10A** | | | **R-15** | | | **R-16** | | | **R-17** | | | **R-19** | | | **R-20** | | | **R-23** | | | **Y-7** | | | **Y-15** | | |  |
| --- | --- | --- | --- | --- | --- | --- | --- | --- | --- | --- | --- | --- | --- | --- | --- | --- | --- | --- | --- | --- | --- | --- | --- | --- | --- | --- | --- | --- | --- | --- | --- | --- | --- | --- | --- | --- | --- |
|  | **V** | **FO** | **FF** | **V** | **FO** | **FF** | **V** | **FO** | **FF** | **V** | **FO** | **FF** | **V** | **FO** | **FF** | **V** | **FO** | **FF** | **V** | **FO** | **FF** | **V** | **FO** | **FF** | **V** | **FO** | **FF** | **V** | **FO** | **FF** | **V** | **FO** | **FF** | **V** | **FO** | **FF** |  |
| **RA (mg 100 g⁻¹ DW)** | 178.6 | 80.1 | 58.1 | 97.2 | 97.7 | 70.6 | 134.8 | 77.8 | 59.4 | 118.8 | 130.8 | 138.2 | 148.7 | 102.8 | 94.6 | 103.7 | 59.0 | 53.8 | 136.9 | 141.8 | 173.6 | 98.7 | 136.6 | 82.8 | 79.8 | 71.8 | 84.5 | 136.2 | 63.2 | 71.3 | 152.8 | 214.6 | 218.3 | 70.6 | 72.3 | 62.3 |  |
| **TPC (mg GAE g⁻¹ DW)** | 10.72 | 8.94 | 5.58 | 7.01 | 10.47 | 7.50 | 8.05 | 8.42 | 7.14 | 6.24 | 9.61 | 9.08 | 9.02 | 8.04 | 5.64 | 8.28 | 7.59 | 7.67 | 8.46 | 12.68 | 13.76 | 11.30 | 14.08 | 7.49 | 7.64 | 4.99 | 8.39 | 8.57 | 9.30 | 6.91 | 11.94 | 14.05 | 20.06 | 7.96 | 9.07 | 10.09 |  |
| **ABTS (µmol TE g⁻¹ DW)** | 268.3 | 254.1 | 192.4 | 189.3 | 219.9 | 179.3 | 205.3 | 192.1 | 217.9 | 182.0 | 265.8 | 231.9 | 233.9 | 207.0 | 145.9 | 227.9 | 203.3 | 201.2 | 204.6 | 332.7 | 306.9 | 347.5 | 363.0 | 220.6 | 185.3 | 172.8 | 216.9 | 245.1 | 224.2 | 204.1 | 288.4 | 366.9 | 372.0 | 224.2 | 251.3 | 251.6 |  |
| **DPPH (µmol TE g⁻¹ DW)** | 72.0 | 67.7 | 49.2 | 39.8 | 91.4 | 60.2 | 43.8 | 78.3 | 77.6 | 43.0 | 53.1 | 77.4 | 64.6 | 63.5 | 49.1 | 65.4 | 61.9 | 69.0 | 60.0 | 142.4 | 117.7 | 88.4 | 115.6 | 59.9 | 51.0 | 36.3 | 65.8 | 63.4 | 81.7 | 57.1 | 88.9 | 86.5 | 182.8 | 48.3 | 80.5 | 85.6 |  |
| **FRAP (µmol TE g⁻¹ DW)** | 156.0 | 133.2 | 89.8 | 110.7 | 147.0 | 135.3 | 114.4 | 125.6 | 111.0 | 100.6 | 163.9 | 147.6 | 137.3 | 122.7 | 91.6 | 119.0 | 116.2 | 118.2 | 124.2 | 199.6 | 226.7 | 186.3 | 223.9 | 123.1 | 105.9 | 86.0 | 136.8 | 137.8 | 132.2 | 118.3 | 166.8 | 234.9 | 339.2 | 116.0 | 132.4 | 136.9 |  |
| *Genotype-specific developmental-stage means at the Bursa site (Year 1); each value is the mean of n = 3 RCBD plot replicates per genotype × stage cell. Rosmarinic acid values were derived from Table S9; ABTS, DPPH, and FRAP values were derived from Table S10. TPC values from the same Bursa Year 1 stage experiment (Folin–Ciocalteu method, see Methods) are presented here.* | | | | | | | | | | | | | | | | | | | | | | | | | | | | | | | | | | | | | |

**Table S12.** Three-way ANOVA summary (genotype × location × year) for total phenolic content (TPC) and antioxidant capacity (ABTS, DPPH, FRAP).

| **Variance source** | **df** | **TPC** | **ABTS** | **DPPH** | **FRAP** |
| --- | --- | --- | --- | --- | --- |
| Genotype (G) | 11 | *** | *** | *** | *** |
| Location (L) | 2 | *** (SS = 46.3%) | *** | *** | *** |
| Year (Y) | 1 | NS (p = 0.165) | *** (Year 2 +34.0%) | *** (Year 2 +76.3%) | *** (Year 2 −4.7%) |
| G × L | 22 | ** | ** | ** | ** |
| G × Y | 11 | ** | ** | ** | ** |
| L × Y | 2 | * | ** | ** | * |
| G × L × Y | 22 | * | * | * | * |

*Statistical analysis: SAS v9.4 (SAS Institute, Cary, NC, USA) PROC GLM (Type III sums of squares); n = 3 RCBD plot replicates per genotype × location × year cell. Mean separation: Tukey's HSD (α = 0.05). Significance codes: *** p < 0.001; ** p < 0.01; * p < 0.05; NS = not significant. SS values reported in the table are those explicitly cited in the manuscript text; the complete ANOVA output (sums of squares, mean squares, F-values, df) is archived in the TÜBİTAK project report (Project No. 111O677, Tables 4.31–4.46).*

**Table S13.** Two-way ANOVA summary (genotype × stage) for the Bursa Year 1 developmental-stage experiment.

| **Variance source** | **df** | **RA** | **TPC** | **ABTS** | **DPPH** | **FRAP** |
| --- | --- | --- | --- | --- | --- | --- |
| Genotype (G) | 11 | *** (SS 50%) | *** (SS 47%) | *** (SS 45%) | *** (SS 43%) | *** (SS 48%) |
| Stage (S) | 2 | ** (SS 6.4%) | ** (SS 5.7%) | * (SS 6.0%) | * (SS 5.9%) | * (SS 6.2%) |
| G × S | 22 | *** (SS 44%) | *** (SS 47%) | *** (SS 49%) | *** (SS 50%) | *** (SS 46%) |

*Bursa site, Year 1 only. Stage units treated as independent observations within each replicate block (destructive harvest; separate plot units assigned to each stage). n = 3 RCBD plot replicates per genotype × stage cell. Statistical analysis: SAS v9.4 PROC GLM. Variance partitioning shows that genotype (G) and genotype × stage (G × S) interactions consistently dominate over stage main effects (S); genotypic rankings for phenolic quality are therefore largely preserved across developmental stages. SS percentages represent the proportion of model sum of squares explained by each fixed effect and interaction, excluding residual error. Significance codes: *** p < 0.001; ** p < 0.01; * p < 0.05.*

**Table S14.** Three-way ANOVA summary (genotype × location × year) for plant height (cm) and fresh herb yield (FHY, kg ha⁻¹).

| **Variance source** | **df** | **Plant height** | **FHY** |
| --- | --- | --- | --- |
| Genotype (G) | 11 | *** | *** |
| Location (L) | 2 | *** | *** |
| Year (Y) | 1 | ** | *** (Year 2 +6.3%) |
| G × L | 22 | ** | ** |
| G × Y | 11 | * | ** |
| L × Y | 2 | * | ** |
| G × L × Y | 22 | NS | * |

*Two-year field experiment (Year 1 = 2013; Year 2 = 2014); n = 3 RCBD plot replicates per genotype × location × year cell. Plant height was recorded on ten representative plants per plot at each of two harvests (flowering onset and full flowering) per season. Fresh herb yield (FHY) was determined as total shoot mass per plot harvested at flowering onset. Statistical analysis: SAS v9.4 (SAS Institute, Cary, NC, USA) PROC GLM (Type III sums of squares). Mean separation: Tukey’s HSD (α = 0.05). Significance codes: *** p < 0.001; ** p < 0.01; * p < 0.05; NS = not significant. The Year 2 cross-location grand mean increase for FHY (+6.3%) was computed from the genotype-level Y1 and Y2 cross-location means reported in Table 2 of the main manuscript; six genotypes increased and six decreased, with magnitudes ranging from −14.8% (R-23) to +31.2% (R-16). Significance levels for plant height and FHY are derived from the agronomic three-way ANOVA archived in the TÜBİTAK 111O677 project report (Tables 4.31–4.32); the complete ANOVA output (sums of squares, mean squares, F-values, df) is available in the project report.*

Table S15. Two-way ANOVA summary (genotype × location) for Year 1 rosmarinic acid and chicoric acid.

| Variance source | df | Rosmarinic acid | Chicoric acid |
| --- | --- | --- | --- |
| Genotype (G) | 11 | *** | *** |
| Location (L) | 2 | *** | *** |
| G × L | 22 | *** | *** |
| Error | 72 |  |  |

Year 1 HPLC–TOF dataset; n = 3 RCBD plot replicates per genotype × location cell. Statistical analysis: SAS v9.4 PROC GLM (Type III sums of squares). Significance codes: *** p < 0.001; ** p < 0.01; * p < 0.05; NS = not significant. This table documents the p-value support for the rosmarinic acid and chicoric acid genotype, location and G × L effects cited in the main manuscript; complete ANOVA output is archived in the TÜBİTAK project report (Project No. 111O677).

**Supplementary Figures**

**Fig. S1.** Two-year means of plant height (cm) and fresh herb yield (FHY, kg ha⁻¹) for twelve *Ocimum basilicum* L. genotypes at three Turkish ecological sites (Bursa, Eskişehir, Tokat). Bars = two-year mean; error bars = ± SD (n = 6 plot replicates per genotype–location). Corresponding data in Table 2 of the main text.


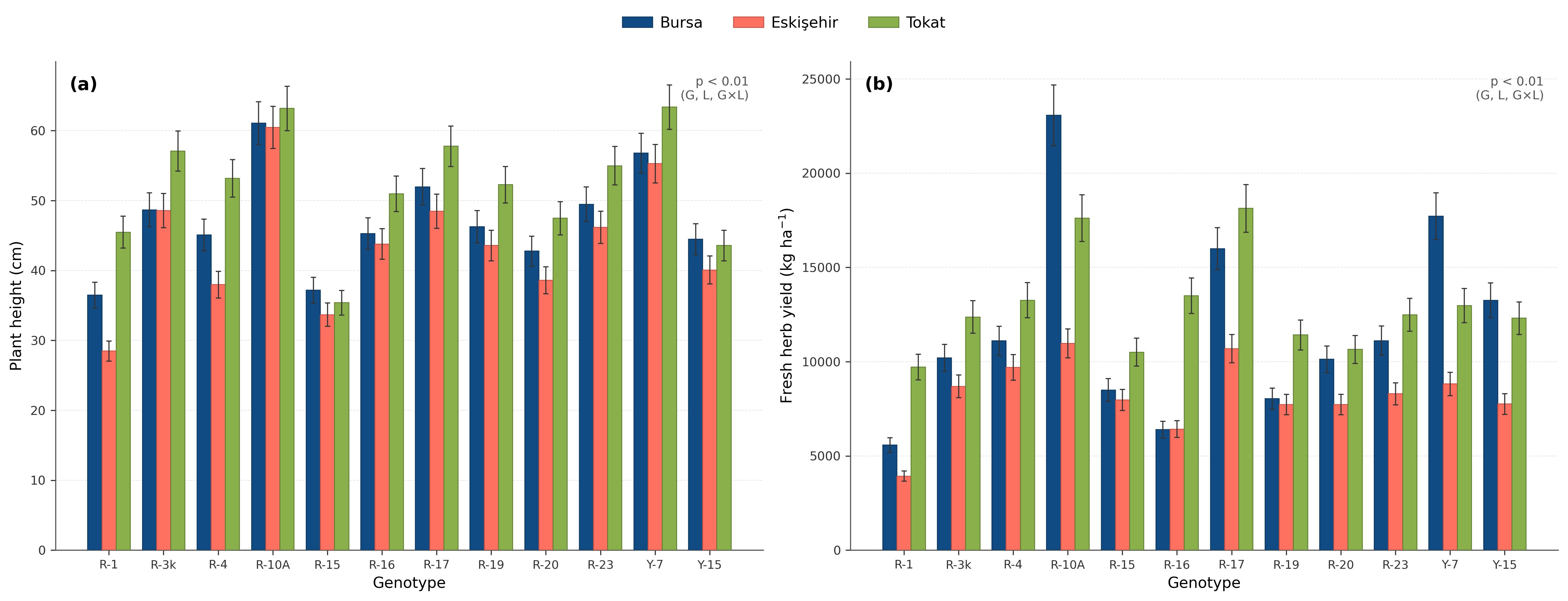


**Fig. S2.** Year 1 means ± SD of (a) rosmarinic acid and (b) chicoric acid (mg 100 g⁻¹ DW) in twelve *Ocimum basilicum* L. genotypes at three Turkish ecological sites (Bursa, Eskişehir, Tokat). Dotted lines indicate location means. Error bars = ± SD (n = 3 RCBD replicate plots). Numerical data in Table 3 of the main text.


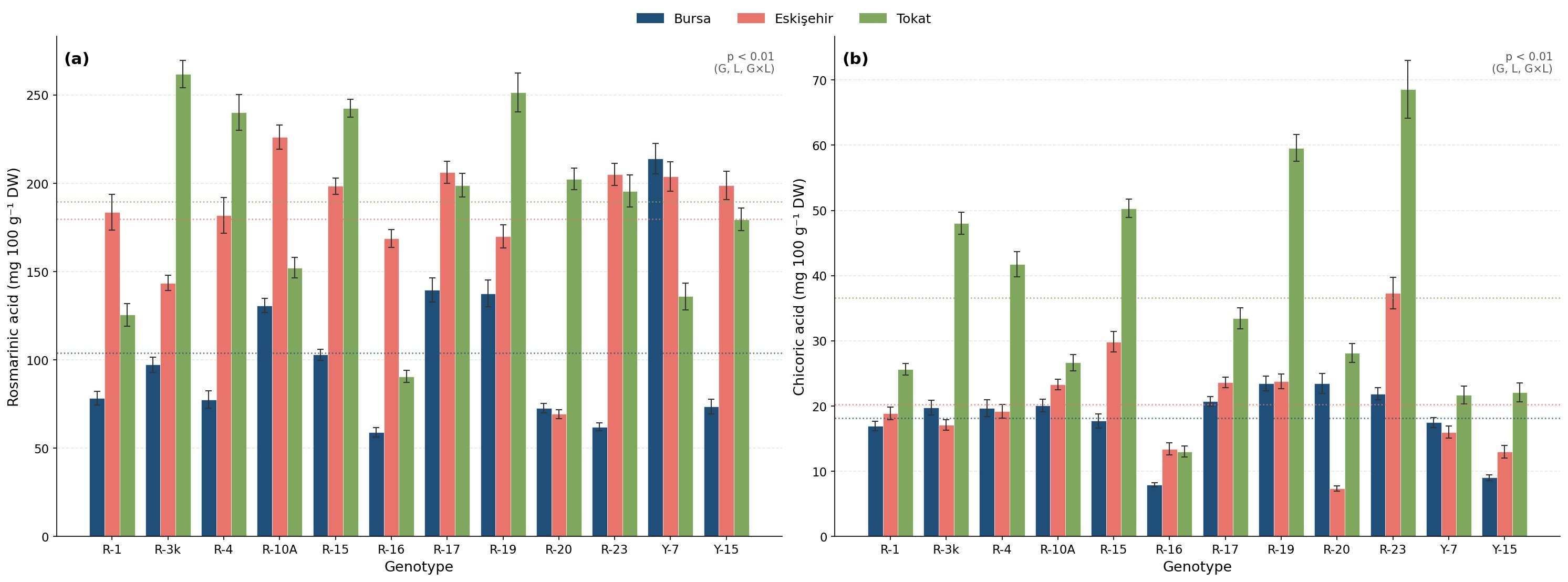

Supplement: Supplementary file 1 — Supplementary Material 1 [file 11130_2026_1512_MOESM2_ESM.docx]
